# One-pot synthesis of (*R*)- and (*S*)-phenylglycinol from bio-based l-phenylalanine by an artificial biocatalytic cascade

**DOI:** 10.1186/s40643-021-00448-5

**Published:** 2021-10-06

**Authors:** Jiandong Zhang, Ning Qi, Lili Gao, Jing Li, Chaofeng Zhang, Honghong Chang

**Affiliations:** 1 Department of Biological and Pharmaceutical Engineering, College of Biomedical Engineering, Taiyuan University of Technology, No. 79 West Yingze Street, Taiyuan, 030024 Shanxi China; 2 College of Environmental Science and Engineering, Taiyuan University of Technology, Taiyuan, Shanxi China

**Keywords:** Cascade biocatalysis, l-Phenylalanine, *Escherichia coli*consortia, Chiral phenylglycinol

## Abstract

Chiral phenylglycinol is a very important chemical in the pharmaceutical manufacturing. Current methods for synthesis of chiral phenylglycinol often suffered from unsatisfied selectivity, low product yield and using the non-renewable resourced substrates, then the synthesis of chiral phenylglycinol remain a grand challenge. Design and construction of synthetic microbial consortia is a promising strategy to convert bio-based materials into high value-added chiral compounds. In this study, we reported a six-step artificial cascade biocatalysis system for conversion of bio-based l-phenylalanine into chiral phenylglycinol. This system was designed using a microbial consortium including two engineered recombinant *Escherichia coli* cell modules, one recombinant *E. coli* cell module co-expressed six different enzymes (phenylalanine ammonia lyase/ferulic acid decarboxylase/phenylacrylic acid decarboxylase/styrene monooxygenase/epoxide hydrolase/alcohol dehydrogenase) for efficient conversion of l-phenylalanine into 2-hydroxyacetophenone. The second recombinant *E. coli* cell module expressed an (*R*)-ω-transaminase or co-expressed the (*S*)-ω-transaminase, alanine dehydrogenase and glucose dehydrogenase for conversion of 2-hydroxyacetophenone into (*S*)- or (*R*)-phenylglycinol, respectively. Combining the two engineered *E*. *coli* cell modules, after the optimization of bioconversion conditions (including pH, temperature, glucose concentration, amine donor concentration and cell ratio), l-phenylalanine could be easily converted into (*R*)-phenylglycinol and (*S*)-phenylglycinol with up to 99% conversion and > 99% *ee*. Preparative scale biotransformation was also conducted on 100-mL scale, (*S*)-phenylglycinol and (*R*)-phenylglycinol could be obtained in 71.0% and 80.5% yields, > 99% *ee*, and 5.19 g/L d and 4.42 g/L d productivity, respectively. The salient features of this biocatalytic cascade system are good yields, excellent *ee*, mild reaction condition and no need for additional cofactor (NADH/NAD^+^), provide a practical biocatalytic method for sustainable synthesis of (*S*)-phenylglycinol and (*R*)-phenylglycinol from bio-based L-phenylalanine.

**Supplementary Information:**

The online version contains supplementary material available at 10.1186/s40643-021-00448-5.

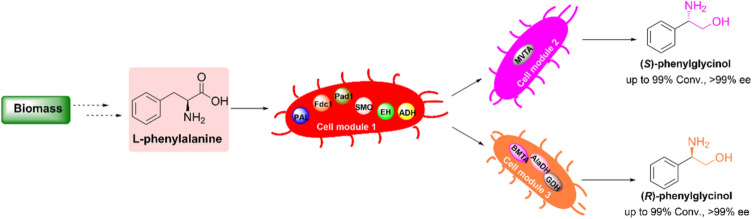

## Introduction

Chiral vicinal amino alcohol moieties are important structural features of many pharmaceutically active molecules and natural products (Bergmeier et al. 2000; Gupta et al. 2018); besides, they can be used as chiral ligands or auxiliaries in asymmetric synthesis (Tan et al. 2017). In this context, enantiopure phenylglycinols are particularly interesting due to their potential use as a key building block for many pharmaceuticals synthesis, such as neurotrophic agent (Panek et al. 1998), h5-HT1D receptor agonists (Russell et al. 1999), antimitotic agents (Kim et al. 2004) and p21-activated kinases (PAK4) inhibitors (Guo et al. 2017).

The industrial synthesis of vicinal amino alcohols typically uses chemical methods. These methods mainly include reduction of amino acids (McKennon et al. 1993; Vandekerkhove et al. 2018), asymmetric ring-opening of epoxides (Overman et al. 1985; Jacobsen et al. 2000), addition of lithiated aziridines to boronic esters (Schmidt et al. 2009), addition of nucleophiles to aminocarbonyls (Reetz et al. 1991) and imines (Kobayashi et al. 1998), and asymmetric aminohydroxylation (AA) of styrene (Li et al. 1996; Sharpless et al. 1975). However, the conventional chemical methods for the synthesis of chiral vicinal amino alcohols are often affected by unsatisfied regioselectivity and enantioselectivity, low product yield, and the toxicity of the metal catalyst and N-protected amino alcohol product. Therefore, the chemical methods used for the industrial synthesis of chiral vicinal amino alcohols are considered to be non-viable for industrial application purposes. However, biocatalysis has been widely used in the synthesis of many useful chemicals, and it is a more sustainable method compared to the above-mentioned methods (You et al. 2021; Wang et al. 2020). In the past two decades, various biocatalytic alternatives have been reported for the synthesis of chiral vicinal amino alcohols, such as racemic amino alcohol kinetic resolution (Wu et al. 2017; Rouf et al. 2011), chiral amino ketones asymmetric reduction (Patel et al. 1993), and α-ketol asymmetric reduction amination (Zhang et al. 2019a; Chen et al. 2019). Recently, two types of biocatalytic cascades for the synthesis of chiral vicinal amino alcohols have been developed via asymmetric ring-opening of epoxides (Zhang et al. 2019b) and asymmetric aminohydroxylation of alkenes (Zhang et al. 2020). The results of these studies showed good conversion percentages (up to 99%) and *ee* values (> 99%) of amino alcohol products from the tested epoxides and alkenes. Although the substrates (i.e., epoxides and alkenes) used in these methods are not expensive and readily available, these substrates are mainly produced from non-renewable carbon resources. Consequently, these methods can be less sustainable compared to other techniques. Therefore, there is a need to find new biocatalytic strategies for synthesizing chiral vicinal amino alcohols from sustainable resources.

l-α-Amino acids are bio-based materials that can be produced from the fermentation of carbohydrates or waste protein sources (Pelckmans et al. 2017). In fact, the annual worldwide amino acids production is about four million tons (Becker et al. 2012). Therefore, the bio-based l-α-amino acids can make a significant contribution to the economic and ecological production of chemical products and materials. In the past decades, several methods have been developed for converting l-α-amino acids into other high added-value compounds (Fotheringham et al. 2006; Studte et al. 2008; Zhang et al. 2019c). For example, cascade biocatalysis processes have been used for conversion of l-phenylalanine into chiral styrene oxides, 1-phenylethane-1,2-diols, mandelic acids and phenylglycines (Zhou et al. 2016). Tan et al. (2021) described an in vivo four-enzyme cascade pathway for efficient conversion of l-tyrosine to d-*p*-hydroxyphenylglycine. For instance, these authors reported d-*p*-hydroxyphenylglycine conversion and *ee* percentages of 92.5% and > 99% from 50 g/L l-tyrosine, respectively (Tan et al. 2021). Moreover, a whole-cell cascade biocatalysis system was established for efficient production of glutarate (yield = 43.8 g/L) from l-lysine with a recombinant *E. coli* microbial consortium by applying a fed-batch strategy (Wang et al. 2019). Recently, Sekar et al. (2020) developed a one-pot cascade biotransformation process for conversion of l-phenylalanine to chiral phenylglycinol for the first time. About 42% conversion of (*R*)-phenylglycinol (99% *ee*) and 26% conversion of (*S*)-phenylglycinol (92% *ee*) were obtained from l-phenylalanine using engineered *E. coli* strains (Sekar et al. 2020). Unfortunately, the low substrate loadings, productivity and *ee* values significantly limit the industrial application of this process. Therefore, there is a need to develop more efficient biocatalysis systems for the synthesis of chiral phenylglycinol from l-phenylalanine.

**Scheme 1:**
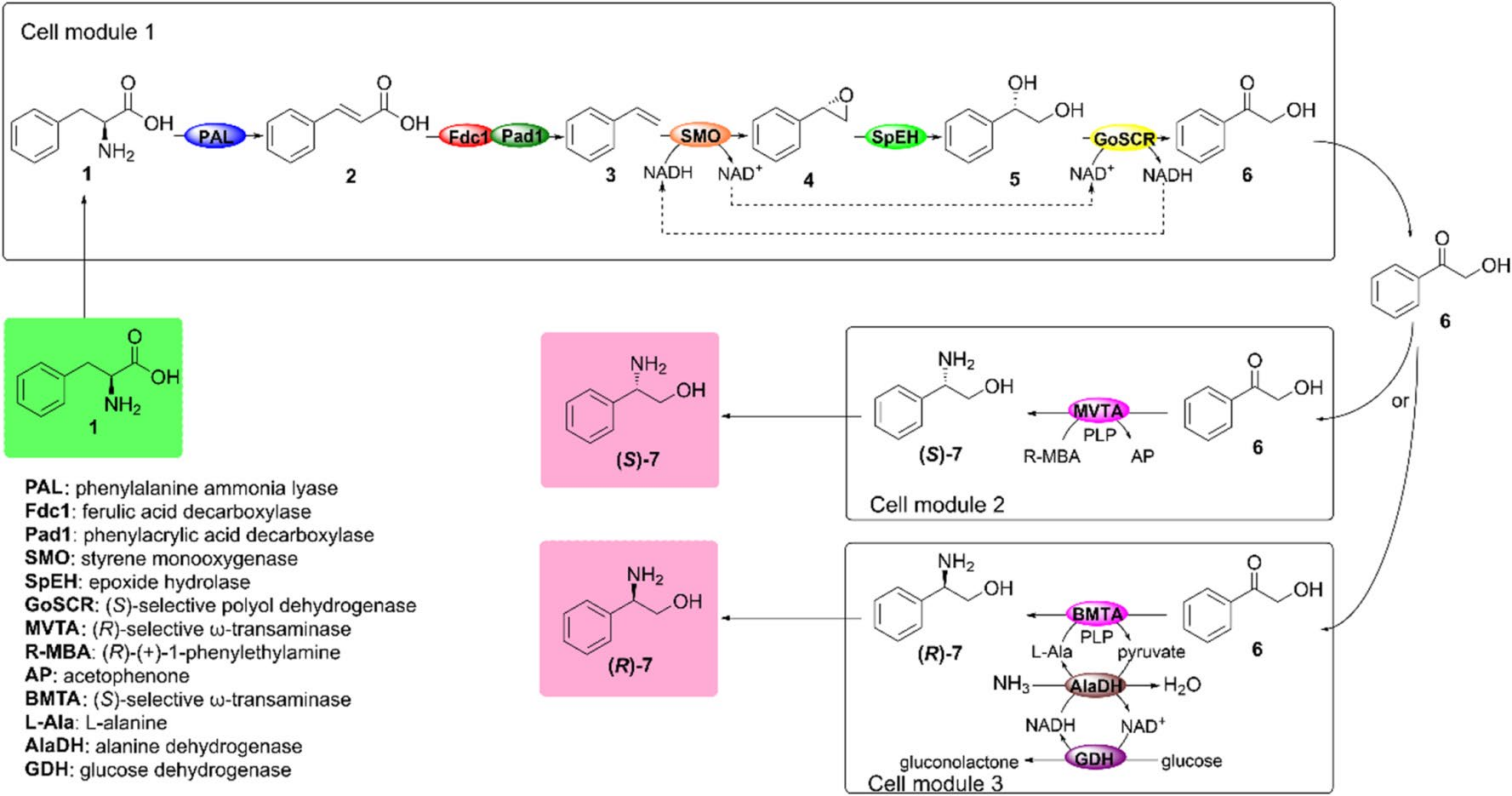
Design and modularization of a de novo designed biocatalytic cascade for production of chiral phenylglycinol from L-phenylalanine. Cell module 1 involves PAL catalyzed deamination of L-phenylalanine **1**, Fdc1 and Pad1 mediates the conversion of *trans*-cinnamic acid **2** to styrene **3**, SMO catalyzed oxidation of styrene **3** to styrene oxide **4**, SpEH catalyzed hydrolysis of styrene oxide **4** to diol **5** and GoSCR catalyzed oxidation of diol **5** to 2-hydroxyacetophenone **6**. Cell module 2 involves MVTA catalyzed asymmetric amination of 2-hydroxyacetophenone **6** to (*S*)-phenylglycinol **7**. Cell module 3 involves BMTA catalyzed asymmetric amination of 2-hydroxyacetophenone **6** to (*R*)-phenylglycinol **7**, as well as AlaDH catalyzed recycling of L-alanine and GDH catalyzed regeneration of NADH cofactor

In this study, a novel artificially designed cascade biocatalysis system was constructed for conversion of bio-based l-phenylalanine into chiral phenylglycinol. This system was designed using a microbial consortium including two recombinant *E. coli* cell modules (Scheme 1). One recombinant *E. coli* cell module co-expressed the following six enzymes: phenylalanine ammonia lyase (PAL), ferulic acid decarboxylase (Fdc1), phenylacrylic acid decarboxylase (Pad1), styrene monooxygenase (SMO), epoxide hydrolase (EH), and alcohol dehydrogenase (ADH). This module was used for achieving an efficient conversion of l-phenylalanine (L-PA, **1**) into 2-hydroxyacetophenone (2-HAP, **6**). The second recombinant *E. coli* cell module expressed the (*R*)-ω-transaminase or co-expressed the (*S*)-ω-transaminase, alanine dehydrogenase, and glucose dehydrogenase for conversion purposes of 2-HAP **6** into (*S*)- or (*R*)-phenylglycinol, respectively. Moreover, when combining these two recombinant *E*. *coli* modules, l-phenylalanine could be easily transformed to (*R*)-phenylglycinol and (*S*)-phenylglycinol with good conversion and excellent *ee* values.

## Materials and methods

### Chemicals

l-Phenylalanine (l-PA), *trans*-cinnamic acid, styrene, (*S*)-1-phenyl-1, 2-ethanediol, styrene oxide, 2-hydroxyacetophenone (2-HAP), (±)-phenylglycinol, (*R*)-phenylglycinol, (*S*)-phenylglycinol, l-alanine (l-Ala), *n*-dodecane, pyridoxal-5′-phosphate (PLP) and (*R*)-α-methylbenzylamine (R-MBA) were from Energy Chemical and Titan Scientific (Shanghai, China). Yeast extract, tryptone and antibiotics (kanamycin, ampicillin and streptomycin) were from Sangon Biotech (Shanghai, China). T4 DNA ligase and restriction enzymes were from New England Biolabs (NEB, Beijing, China). Isopropyl β-d-1-thiogalactopyranoside (IPTG) and Taq plus DNA polymerase were purchased from Tsingke (Beijing, China). All other chemical reagents were obtained from commercial sources.

### Plasmids, microorganisms and media

The expression plasmids (pET28a (+), pETduet-1, pRSFduet-1 and pCDFduet-1) were from Novagen (Madison, WI, USA). Previously constructed recombinant plasmids (pET28a-SMO, pETduet-SpEH, pET28a-GoSCR, pET28a-MVTA, pET28a-BMTA and pETduet-GDH) (Zhang et al. 2020) were stored in our lab. The host strain *E. coli* T7 supercompetent cells were purchased from NEB (Beijing, China). The *E. coli* strains were grown at 37 °C in Luria–Bertani (LB) medium or Terrific Broth (TB) medium. Antibiotics ampicillin (0.1 mg/mL), kanamycin (0.05 mg/mL) and streptomycin (0.1 mg/mL) were utilized for the selection of recombinant *E. coli* cells. *Bacillus subtilis* sp. 168 stored in our lab was maintained on MRS agar slants and grown in MRS medium at 30 °C.

### Construction of the recombinant *E. coli* strains

All the primers used in this study were synthesized by Tsingke (Beijing, China) and listed in Additional file 1: Table S1. All the constructed plasmids and recombinant *E. coli* cells are listed in Additional file 1: Table S2. *E. coli* (GoSCR), *E. coli* (GDH) (Cui et al. 2017), *E. coli* (MVTA) (Zhang et al. 2019a), *E. coli* (SpEH) (Zhang et al. 2019b), *E. coli* (SMO) and *E. coli* (BMTA) (Zhang et al. 2020) were constructed as described previously.

Regarding the recombinants *E. coli* (PAL), *E. coli* (Fdc1) and *E. coli* (Pad1), the PAL gene from *Arabidopsis thaliana* (Cochrane et al. 2004) and the Fdc1 and Pad1 genes from *Aspergillus niger* (Payne et al. 2015) were synthesized by Tsingke (Beijing, China). The DNA fragments of PAL, Fdc1, and Pad1 were then amplified by polymerase chain reaction (PCR) using the primers listed in Additional file 1: Table S1. After, the PCR products were isolated, double digested with the restriction endonucleases (NdeI and XhoI), and ligated into pET28a (+), which were further digested with the same restriction enzymes. The resulting recombinant plasmids (pET28a-PAL, pET28a-Fdc1 and pET28a-Pad1) were separately transformed into the competent *E. coli* T7 to form *E. coli* (PAL), *E. coli* (Fdc1), and *E. coli* (Pad1).

For the design of *E. coli* (AlaDH), the genome DNA was extracted from *Bacillus subtilis* sp. 168 by using a Bacterial DNA extraction kit supplied by Sangon Biotech (China). After, the DNA fragment of the AlaDH (alanine dehydrogenase) gene was amplified from the genomic DNA of *Bacillus subtilis* sp. 168 with PCR using the primers shown in Additional file 1: Table S1. The PCR product was isolated and double digested with BamHI and PstI. After, it was ligated into the expression vector pETduet-1 at the BamHI/PstI sites to form the recombinant pETduet-AlaDH. The constructed recombinant plasmid pETduet-AlaDH was then transformed into the competent *E. coli* T7 to form the recombinant *E. coli* (pETduet-AlaDH). In addition, the recombinant *E. coli* (pCDFduet-AlaDH) was constructed using a similar procedure.

Moreover, the recombinant *E. coli* (Fdc1-Pad1) was designed by amplifying the DNA fragments of Fdc1 and Pad1 with PCR using the primers listed in Additional file 1: Table S1. The Fdc1 gene was double-digested with the BamHI and NotI and ligated into the pRSFduet-1 at the BamHI/NotI sites to form the recombinant pRSFduet-Fdc1. In addition, the Pad1 gene was double-digested with the restriction endonucleases NdeI and XhoI and ligated into pRSFduet-Fdc1 at the BamHI/XhoI sites to form the pRSFduet-Fdc1-Pad1 (named as RFP). Furthermore, the recombinant plasmid pRSFduet-Fdc1-Pad1 was then transformed into the competent *E. coli* T7 to form the recombinant *E. coli* (pRSFduet-Fdc1-Pad1) cells, designated as *E. coli* (RFP). Furthermore, the recombinant *E. coli* (pETduet-Fdc1-Pad1) (designated as *E. coli* (DFP)) and *E. coli* (pCDFduet-Fdc1-Pad1) (designated as *E. coli* (CFP)) were constructed in a similar way.

In the case of *E. coli* (pETduet-SpEH-PAL), the DNA fragments of both epoxide hydrolase (SpEH) and PAL enzymes were amplified with PCR using the primers shown in Additional file 1: Table S1. The SpEH gene was double-digested with the restriction endonucleases BamHI and HindIII and ligated into the pETduet-1 at the BamHI/HindIII sites to form the recombinant pETduet-SpEH. Also, the PAL gene was double-digested with NdeI and XhoI and ligated into the pETduet-SpEH at the NdeI/XhoI sites to form the recombinant pETduet-SpEH-PAL (known as DEA). In addition, the recombinant plasmid pETduet-SpEH-PAL was transformed into the competent *E. coli* T7 to form *E. coli* (pETduet-SpEH-PAL) which was named *E. coli* (DEA).

Moreover, the following recombinants were constructed in a similar way: *E. coli* (pCDFduet-PAL-SMO) (designated as *E. coli* (CAS)), *E. coli* (pRSFduet-SpEH-GoSCR) (named as *E. coli* (REG)), *E. coli* (pCDFduet-GoSCR-SMO) (known as *E. coli* (CGS)), *E. coli* (pETduet-SpEH-SMO) (designated as *E. coli* (DES)), and *E. coli* (pRSFduet-GoSCR-PAL) (named as *E. coli* (RGA)). The enzyme GoSCR referred to NAD^+^-dependent alcohol dehydrogenase.

Regarding the recombinant *E. coli* (PAL-Fdc1-Pad1-SMO-SpEH-GoSCR), the constructed recombinant plasmids pRSFduet-Fdc1-Pad1, pETduet-SpEH-PAL, and pCDFduet-GoSCR-SMO had different antibiotic resistance genes. These recombinants were simultaneously transformed into the competent *E. coli* T7 to form the recombinant *E. coli* (PAL-Fdc1-Pad1-SMO-SpEH-GoSCR) cells, which were designated as *E. coli* (RFP-DEA-CGS). Furthermore, the recombinant *E. coli* (DFP-CAS-REG) and *E. coli* (CFP-DES-RGA) cells were similarly constructed with different recombinant plasmid combinations. These recombinants co-expressed PAL, Fdc1, Pad1, SMO, SpEH, and GoSCR.

For *E. coli* (pETduet-GDH-AlaDH), the GDH and AlaDH genes were amplified by PCR using the primers shown in Additional file 1: Table S1. After, the GDH (glucose dehydrogenase) gene was double-digested with BamHI and HindIII and ligated into the pETduet-1 at the BamHI/HindIII sites to form the recombinant pETduet-GDH. Then, the AlaDH gene was double-digested with the restriction endonucleases KpnI and XhoI and further ligated into the pETduet-GDH at the KpnI/XhoI sites to obtain a new recombinant plasmid pETduet-GDH-AlaDH (known as DGA). This recombinant plasmid was then transformed into the competent *E. coli* T7 to form the recombinant *E. coli* (pETduet-GDH-AlaDH), designated as *E. coli* (DGA).

For *E. coli* (BMTA-GDH-AlaDH), the constructed recombinant plasmids pET28a-BMTA and pETduet-GDH-AlaDH were simultaneously transformed into the competent *E. coli* T7 to form the recombinant *E. coli* (BMTA-GDH-AlaDH), designated as *E. coli* (EB-DGA). These two recombinant plasmids had different antibiotic resistance genes. Similarly, the recombinant plasmids pET28a-BMTA, pETduet-GDH, and pCDFduet-AlaDH were simultaneously transformed into the competent *E. coli* T7 to form the recombinant *E. coli* (BMTA-GDH-AlaDH), named *E. coli* (EB-DG-CA). The enzyme BMTA refers to (*S*)-selective ω-transaminase.

The expression of all genes from the above-constructed recombinant *E. coli* strains was confirmed by sodium dodecyl sulfate polyacrylamide gel electrophoresis (SDS-PAGE) and testing the activity of corresponding enzymes.

### Enzyme expression and activity analysis

The constructed recombinant *E. coli* cells were cultivated at 37 °C for 12 h in 5 mL LB medium (10 g/L peptone, 5 g/L yeast extract and 10 g/L NaCl, pH7.0) containing appropriate antibiotics. Two milliliter of seed culture was then inoculated into 100 mL of Terrific Broth (TB) medium (12 g/L tryptone, 24 g/L yeast extract, 4 g/L glycerol, 17 mM KH_2_PO_4_, 72 mM K_2_HPO_4_) supplemented with the appropriate antibiotics, and cultivated at 37 °C for 2–3 h. When OD_600_ value of the recombinant *E. coli* cells reached 0.6–0.8, IPTG (0.5 mM) was then added to the medium to induce the protein expression. After incubation for 12–20 h at 20 °C and 200 rpm, cells were harvested by centrifugation (8000 rpm) at 4 °C for 10 min, washed twice with cold saline and resuspended in sodium phosphate buffer (100 mM, pH 7.5). The cell pellets were put on ice and lysed by ultra-sonication for 90 times at 400 W for 4 s with 6 s of interval. The cell debris were removed by centrifugation (12,000 rpm, 30 min) at 4 °C, and the soluble portion of cell lysate was stored at − 80 °C for further use.

The activity of phenylalanine ammonia lyase (PAL) was measured as described previously (Cochrane et al. 2004). The reaction mixture consisted of 100 mM sodium phosphate buffer (pH 8.5), 5 mM l-phenylalanine substrates, and an appropriate amount of enzyme in a total volume of 0.5 mL. The reaction mixture was incubated at 30 °C for 5 min. The reaction samples (100 μL) were mixed with acetonitrile (900 μL) containing 2 mM acetophenone (internal standard), centrifuged at 12,000 rpm for 10 min. The concentration of the *trans*-cinnamic acid was determined by HPLC analysis. 1 unit of activity refers to the amount of catalyst that catalyzed the conversion of 1 μmol l-phenylalanine to *trans*-cinnamic acid per min.

The activity of decarboxylases (Fdc1 and Pad1) was measured as described previously (Payne et al. 2015). The reaction mixture consisted of 100 mM sodium phosphate buffer (pH 7.0), 5 mM *trans*-cinnamic acid, and an appropriate amount of enzyme in a total volume of 0.5 mL. The reaction mixture was incubated at 30 °C for 5 min, the reaction samples (100 μL) were mixed with acetonitrile (900 μL) containing 2 mM acetophenone (internal standard), centrifuged at 12,000 rpm for 10 min. The concentration of the styrene was determined by HPLC analysis. 1 unit of activity refers to the amount of catalyst that catalyzed the conversion of 1 μmol *trans*-cinnamic acid to styrene per minute.

The activity of styrene monooxygenase (SMO) was measured as described previously (Xu et al. 2009). The reaction mixture consisted of 100 mM sodium phosphate buffer (pH 7.0), 5 mM styrene substrate, 1 mM NADH, and an appropriate amount of enzyme in a total volume of 0.5 mL. After incubation at 30 °C for 5 min, the reaction samples (300 µL) were saturated with sodium chloride, and extracted with ethyl acetate (EtOAc) containing 5 mM of *n*-dodecane (internal standard). The organic phase was dried over anhydrous Na_2_SO_4_, and the concentration of styrene oxide was determined by GC analysis. 1 unit of activity refers to the amount of catalyst that catalyzed the conversion of 1 μmol styrene to styrene oxide per minute.

The activity of epoxide hydrolase (SpEH) was measured as described previously (Wu et al. 2013). The reaction mixture consisted of 100 mM sodium phosphate buffer (pH 7.0), 10 mM styrene oxide substrate, and an appropriate amount of enzyme in a total volume of 0.5 mL. After incubation at 30 °C for 5 min, the reaction samples (300 µL) were saturated with sodium chloride, and extracted with EtOAc containing 5 mM of *n*-dodecane (internal standard). The organic phase was dried over anhydrous Na_2_SO_4_, and the concentration of the 1-phenyl-1,2-ethanediol was determined by GC analysis. 1 unit of activity refers to the amount of catalyst that catalyzed the conversion of 1 μmol styrene oxide to 1-phenyl-1,2-ethanediol per minute.

The oxidation activity of alcohol dehydrogenase was measured as described previously (Zhang et al. 2020). The reaction mixture consisted of 100 mM sodium phosphate buffer (pH 7.5), 0.2 mM NAD^+^, 10 mM 1-phenyl-1,2-ethanediol and an appropriate amount of enzyme in a total volume of 1.0 mL, the reaction was performed at 30 °C for 1 min. 1 unit of activity refers to the amount of catalyst that catalyzed the conversion of 1 μmol NAD^+^ to NADH per minute.

The activity of alanine dehydrogenase (AlaDH) was measured as described previously (Lerchner et al. 2016). The reaction mixture consisted of 100 mM sodium phosphate buffer (pH 7.5), 0.2 mM NADH, 5 mM pyruvate, 200 mM NH_4_Ac and an appropriate amount of enzyme in a total volume of 1.0 mL. The reaction was performed at 30 °C for 1 min. 1 unit of activity refers to the amount of catalyst that catalyzed the conversion of 1 μmol NADH to NAD^+^ per minute.

The activity of transaminase was assayed as described in our previous study (Zhang et al. 2017). The activity of glucose dehydrogenase (GDH) was measured as described in our previous study (Cui et al. 2017).

The activity of recombinant *E. coli* (DFP-CAS-REG) cells was measured by testing the formation of 2-HAP **6**. The reaction mixture consisted of 100 mM sodium phosphate buffer (pH 7.5), 10 mM L-PA **1**, 10 mM glucose and 5 g cell dry weight (cdw)/L recombinant *E. coli* cells in a total volume of 1.0 mL, the reaction was performed at 25 °C and 200 rpm for 10 min. The concentration of 2-HAP was determined by GC analysis. 1 unit of activity refers to the amount of catalyst that catalyzed the conversion of 1 μmol L-PA to 2-HAP per min.

The protein concentration was determined by the Bradford method (Bradford 1975).

### In vitro conversion of L-PA 1 into 2-HAP 6 and chiral phenylglycinol 7

The freshly prepared recombinant *E. coli* cells (*E. coli* (PAL), *E. coli* (Fdc1), *E. coli* (Pad1), *E. coli* (SMO), *E. coli* (SpEH), *E. coli* (GoSCR), *E. coli* (BMTA) and *E. coli* (MVTA)) were washed twice with sterile deionized water, and resuspended in sterile deionized water to an OD_600_ of 30, respectively. The cell pellets were put on ice and lysed by ultra-sonication for 90 times at 400 W for 4 s with 6 s of interval. After centrifugation for 30 min at 4 °C and 12,000 rpm, the soluble portion of cell lysate was frozen at − 80 °C overnight. Then the frozen cell-free extracts were lyophilized for 48 h using a vacuum freeze dryer (SCIENTA-10, China) at a temperature of − 50 °C and a vacuum of 0.055 mbar. For conversion of L-PA **1** to 2-HAP **6**, the reaction mixture consisted of 100 mM sodium phosphate buffer (pH 7.5), 10–20 mM L-PA, 0.5 mM NADH, 15 mg/mL PAL, 15 mg/mL Fdc1, 15 mg/mL Pad1, 20 mg/mL SMO, 10 mg/mL SpEH, 20 mg/mL GoSCR in a total volume of 5 mL. For conversion of L-PA **1** to phenylglycinol **7**, the reaction mixture consisted of 100 mM sodium phosphate buffer (pH 7.5), 10–20 mM L-PA **1**, 0.5 mM NADH, 15 mg/mL PAL, 15 mg/mL Fdc1, 15 mg/mL Pad1, 20 mg/mL SMO, 10 mg/mL SpEH, 20 mg/mL GoSCR, 15 mg/mL MVTA or 15 mg/mL BMTA, 0.1 mM PLP, and 15–25 mM (*R*)-MBA or 200 mM L-Ala in a total volume of 5 mL. The reactions were conducted at 30 °C and 200 rpm. At appropriate intervals, samples were taken for GC analysis.

### In vivo conversion of L-PA 1 into 2-HAP 6 with the resting cells of recombinant *E. coli* cells

The freshly prepared recombinant *E. coli* (RFP-DEA-CGS), *E. coli* (DFP-CAS-REG) and *E. coli* (CFP-DES-RGA) cells were washed twice with sterile deionized water and frozen at − 80 °C overnight. Then the frozen cells were lyophilized for 48 h using the vacuum freeze dryer at a temperature of − 50 °C and a vacuum of 0.055 mbar. The reaction mixture consisted of 100 mM sodium phosphate buffer (pH 7.5), 10 g cdw/L lyophilized recombinant *E. coli* cells, 10 mM L-PA **1** and 10 mM glucose in a total volume of 5 mL. The reactions were performed at 25 °C and 200 rpm. At appropriate intervals, samples were taken for GC analysis.

### In vivo conversion of 2-HAP 6 into (*S)*- or (*R*)-phenylglycinol 7

The freshly prepared recombinant *E. coli* (MVTA) and *E. coli* (EB-DGA) cells were washed twice with sterile deionized water and frozen at − 80 °C overnight and lyophilized for 48 h using the vacuum freeze dryer at a temperature of − 50 °C and a vacuum of 0.055 mbar. The reaction mixture consisted of 100 mM sodium phosphate buffer (pH 8.0), 10 g cdw/L lyophilized recombinant *E. coli* cells, 10 mM 2-HAP **6**, 0.1 mM PLP, 10% DMSO, 20 mM R-MBA or 400 mM L-Ala (including 150 mM NH_3_/NH_4_Cl) in a total volume of 5 mL. The reactions were performed at 30 °C and 200 rpm. At appropriate intervals, samples were taken for GC analysis.

### Conversion of L-PA 1 into (*S*)-phenylglycinol 7 with the mixture of resting cells of *E. coli* (RFP-DEA-CGS) and *E. coli* (MVTA)

The reaction mixture consisted of 100 mM sodium phosphate buffer (pH 8.0), 20 g cdw/L lyophilized recombinant *E. coli* (RFP-DEA-CGS) cells, 15 g cdw/L lyophilized recombinant *E. coli* (MVTA) cells, 10–50 mM L-PA **1**, 5–40 mM glucose, 0.1 mM PLP, 25–40 mM (*R*)-MBA in a total volume of 5 mL. The reactions were performed at 25 °C and 200 rpm. At appropriate intervals, samples were taken for GC analysis.

### Conversion of L-PA 1 into (*R*)-phenylglycinol 7 with the mixture of resting cells of *E. coli* (RFP-DEA-CGS) and *E. coli* (EB-DGA)

The reaction mixture consisted of 100 mM sodium phosphate buffer (pH 8.0), 15 g cdw/L lyophilized recombinant *E. coli* (RFP-DEA-CGS) cells, 20 g cdw/L lyophilized recombinant *E. coli* (EB-DGA) cells, 10–50 mM L-PA **1**, 5–40 mM glucose, 0.1 mM PLP, 100–600 mM L-Ala and 0–200 mM NH_3_/NH_4_Cl in a total volume of 5 mL. The reactions were performed at 25 °C and 200 rpm. At appropriate intervals, samples were taken for GC analysis.

### Preparation experiment

For preparation of (*S*)-phenylglycinol **7**, the reaction was conducted in 100 mL sodium phosphate buffer (100 mM, pH 7.5) containing 20 mM (330.4 mg) L-PA **1**, 20 g cdw/L *E. coli* (RFP-DEA-CGS), 15 g cdw/L *E. coli* (MVTA), 10 mM glucose, 0.1 mM PLP and 30 mM (*R*)-MBA. For preparation of (*R*)-phenylglycinol **7**, the reaction was conducted in 100 mL sodium phosphate buffer (100 mM, pH 7.5) containing 20 mM (330.4 mg) L-PA **1**, 15 g cdw/L *E. coli* (RFP-DEA-CGS), 20 g cdw/L *E. coli* (EB-DGA), 20 mM glucose, 0.1 mM PLP, 400 mM L-Ala and 150 mM NH_3_/NH_4_Cl. The reactions were performed at 25 °C and 200 rpm for 12 h. After the reactions were completed, the reaction mixtures were basified (pH > 10) by adding NaOH (10 N), and extracted with EtOAc for three times (3 × 50 mL). The combined organic phases were dried over anhydrous Na_2_SO_4_. The solvent was removed by evaporation and the residue was purified using a silica gel column with EtOAc/methanol (10:1) as eluent.

(*S*)-**7** was obtained as a white solid in 71.0% yield (194.8 mg) and > 99% *ee*. ^1^H NMR (400 MHz, 298 K, CDCl_3_) δ_H_ 7.34–7.26 (5H, m, Ph), 4.02 (1H, dd, ^3^*J*_HH_ = 4.0 Hz, 8.5 Hz, CH), 3.73–3.70 (1H, dd, ^2^*J*_HH_ = 11.0 Hz, ^3^*J*_HH_ = 4.0 Hz, CH_2_), 3.56–3.52 (1H, dd, ^2^*J*_HH_ = 11.0 Hz, ^3^*J*_HH_ = 8.5 Hz, CH_2_), 2.45 (br, 3H).

(*R*)-**7** was obtained as a yellow oil in 80.5% yield (220.9 mg) and > 99% *ee*. ^1^H NMR (400 MHz, 298 K, CDCl_3_) δ_H_ 7.37–7.26 (5H, m, Ph), 4.07–4.03 (1H, dd, ^3^*J*_HH_ = 4.0 Hz, 8.5 Hz, CH), 3.76–3.72 (^1^H, dd, ^2^*J*_HH_ = 11.0 Hz, ^3^*J*_HH_ = 4.0 Hz, CH_2_), 3.58–3.54 (1H, dd, ^2^*J*_HH_ = 11.0 Hz, ^3^*J*_HH_ = 8.5 Hz, CH_2_), 2.40 (br, 3H).

### Assay methods

For GC analysis of styrene **3**, (*S*)-1-phenyl-1, 2-ethanediol **5** and 2-HAP **6**, the reaction samples (300 µL) were saturated with sodium chloride, mixed with 300 µL of EtOAc containing 20 mM of *n*-dodecane (internal standard), after centrifugation (12,000 rpm) at 4 °C for 5 min, the organic phase was separated and dried over anhydrous Na_2_SO_4_. The concentration of styrene, (*S*)-1-phenyl-1, 2-ethanediol and 2-HAP was determined by GC analysis. For GC analysis of phenylglycinol **7**, the reaction samples (300 µL) were basified (pH > 10) by adding NaOH (10 N), saturated with sodium chloride, and extracted with EtOAc (300 µL) containing 20 mM of *n*-dodecane (internal standard). The organic phase was dried over anhydrous Na_2_SO_4_, the concentration of the phenylglycinol **7** was determined by GC analysis. GC analysis was conducted using Shimadzu GC-14C gas chromatography system with a flame ionization detector. The column was an Agilent HP-5 (30 m × 0.32 mm × 0.25 mm). Parameter: injector temperature, 250 °C; detector temperature, 275 °C; column temperature: 120 °C. The enantiomeric excess of (*S*)-phenylglycinol or (*R*)-phenylglycinol was determined by chiral GC as described previously (Zhang et al. 2020).

## Results and discussion

### Design and in vitro construction of the artificial biocatalytic cascade for conversion of L-PA 1 into chiral phenylglycinol 7

A sequential cascade biocatalysis was designed by retrosynthetic strategy to convert bio-based L-PA **1** into chiral phenylglycinol **7** (Scheme 1). First, L-PA **1** was deaminized to *trans*-cinnamic acid **2** by the PAL enzyme, followed by oxidative decarboxylation of *trans*-cinnamic acid **2** to styrene **3** by PAD. Moreover, the styrene **3** compound was subsequently oxidized to styrene oxide **4** by SMO, and styrene oxide **4** was then hydrolyzed to 1-phenyl-1,2-ethanediol **5** by EH. This 1-phenyl-1,2-ethanediol **5** compound was oxidized to 2-HAP **6** by ADH. Finally, an asymmetric reduction amination converted the 2-HAP **6** to chiral phenylglycinol **7** by an ω-transaminase (ωTA).

Moreover, PAL from *Arabidopsis thaliana* (Cochrane et al. 2004), which plays an important role in the process of ammonia removal, was selected for converting L-PA **1** into *trans*-cinnamic acid **2** for in vitro reconstruction of the biocatalysis cascade. In the second decarboxylation step, Fdc1 and Pad1 from *Aspergillus niger* were selected for conversion of *trans*-cinnamic acid **2** into styrene **3**. In fact, both enzymes can work together more efficiently in the decarboxylation process of the former compound (Payne et al. 2015). In the third step, the NADH-dependent SMO from *Pseudomonas* sp. VLB120 was used for converting styrene **3** into (*S*)-styrene epoxide **4** (Xu et al. 2009). Furthermore, in the fourth step, SpEH from *Sphingomonas* sp.HXN-200 was used for selective hydrolysis of **4** to (*S*)-diol **5** (Wu et al. 2013). After, in the fifth step, the GoSCR enzyme from *Gluconobacter oxydans* 621H (Cui et al. 2017) was selected for oxidation of (*S*)-diol **5** into 2-HAP **6**. In the final amination step, an (*R*)-selective ω-transaminase (MVTA) from *Mycobacterium vanbaalenii* (Zhang et al. 2019) and the BMTA enzyme from *Bacillus megaterium* SC6394 (Zhang et al. 2020) were then employed to convert 2-HAP **6** to (*S*)-**7** and (*R*)-**7**, respectively. The NAD^+^ and NADH recycling system was generated in the third and fifth step of the catalytic process. All the enzymes used in this study were lyophilized cell-free extracts.

In addition, the activities of these lyophilized enzymes were tested considering their corresponding substrates (Additional file 1: Table S3). After, the L-PA (10–20 mM) and NADH (0.5 mM) compounds were added into the reaction system including a set of six different enzymes (PAL, Fdc1, Pad1, SMO, SpEH, and GoSCR). Interestingly, 2-HAP **6** was obtained with a 99% conversion percentage from 10–20 mM L-PA without optimizing the reaction conditions (Table 1). In addition, the (*R*)-**7** and (*S*)-**7** compounds were obtained with an *ee* value of > 99% and between a range of conversion percentages from 66.8 to 87.2% from 10 to 20 mM L-PA with the mixture of seven enzymes (PAL, Fdc1, Pad1, SMO, SpEH, GoSCR, and MVTA or BMTA) (Table 1). These results showed that the designed cascade biocatalysis complex was successfully in vitro constructed for conversion of L-PA **1** into chiral **7**. However, the in vitro cascade reactions needed the application of multiple enzymes. In addition, the cell-free enzymes were easily affected by a rapid deactivation, and the redox reaction required an expensive cofactor (NAD^+^/NADH), which can increase the reaction costs. Consequently, it was decided to construct the recombinant *E. coli* whole cells that were able to co-express the necessary enzymes for conversion of L-PA **1** into chiral phenylglycinol **7**.

**Table 1:** Cascade biocatalysis for conversion of L-PA 1 to 2-HAP 6 and chiral phenylglycinol 7 with the mixture of lyophilized cell-free extract^a^

Entry	Sub. (mM)	Time (h)	Yield of 5 (%)^d^	Yield of 6 (%)^d^	Yield of 7 (%)^d^	*ee* of 7 (%)^e^
1	10^b^	7	< 1	99	–	–
2	20^b^	12	< 1	99	–	–
3	10^c^	12	16.1	< 1	83.0	> 99 (R)
4	20^c^	24	32.5	< 1	66.8	> 99 (R)
5	10^c^	12	2.6	10.2	87.2	> 99 (S)
6	20^c^	24	4.3	25.3	70.4	> 99 (S)

^a^The reactions were conducted in 5 mL sodium phosphate buffer (100 mM, pH 7.5)

^b^ The reactions containing 10–20 mM L-PA, 0.5 mM NADH, 15 mg/mL PAL, 15 mg/mL Fdc1, 15 mg/mL Pad1, 20 mg/mL SMO, 10 mg/mL SpEH, 20 mg/mL GoSCR, at 30 °C and 200 rpm for 6 h

^c^ The reactions containing 10–20 mM L-PA, 0.5 mM NADH, 15 mg/mL PAL, 15 mg/mL Fdc1, 15 mg/mL Pad1, 20 mg/mL SMO, 10 mg/mL SpEH, 20 mg/mL GoSCR, 15 mg/mL MVTA or 15 mg/mL BMTA, 0.1 mM PLP, (*R*)-MBA (15–25 mM) or L-alanine (200 mM), at 30 °C and 200 rpm for 6 h

^d^ Yield was determined by GC

^e^ *ee* was determined by chiral GC

### Construction of the recombinant *E. coli* cell modules for conversion of L-PA into 2-HAP

Co-expression of multi-enzymes in one microbial cell will usually result in serious protein expression burden and redox constraints, but these negative factors can be reduced by distributing the biocatalytic pathway among different cell modules (Wang et al. 2020). Therefore, a synthetic microbial consortium was designed and constructed to convert L-PA **1** into chiral phenylglycinol **7**. This microbial consortium included two different recombinant *E. coli* cell modules (Scheme 1). Therefore, one recombinant *E. coli* cell module co-expressed the PAL/Fdc1/Pad1/SMO/EH/GoSCR enzymatic set for converting L-PA **1** into 2-HAP **6**. The second recombinant *E. coli* cell module expressed the MVTA enzyme or co-expressed the BMTA/AlaDH/GDH enzymes set for converting 2-HAP into (*S*)- or (*R*)-phenylglycinol **7**, respectively.

**Fig. 1:**
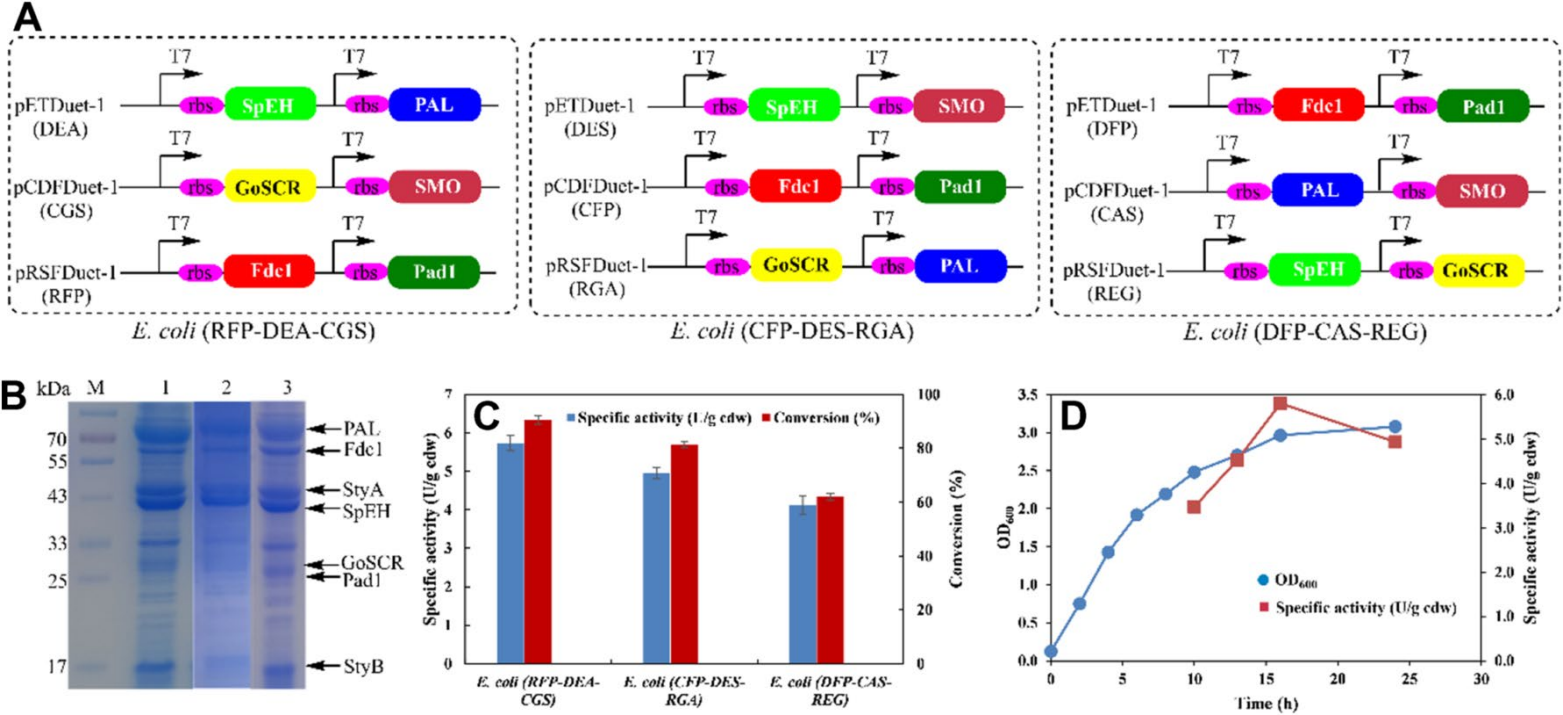
Construction of recombinant *E. coli* cells co-expression of six enzymes for conversion of L-PA **1** to 2-HAP **6**. **A** Construction of recombinant *E. coli* cells co-expression of six enzymes based on different plasmid configurations. **B** SDS-PAGE analysis of recombinant *E. coli* cells. M: marker; lane 1: cell-free extract of *E. coli* (RFP-DEA-CGS), lane 2: cell-free extract of *E. coli* (CFP-DES-RGA); lane 3: cell-free extract of *E. coli* (DFP-CAS-REG). **C** Activities of three different recombinant *E. coli* cells for conversion of L-PA **1** to 2-HAP **6**. **D** Cell growth and specific activity of *E. coli* (RFP-DEA-CGS) for conversion of L-PA **1** to 2-HAP **6**

Additionally, a recombinant *E. coli* cells module **1** that co-expressed six enzymes (PAL, Fdc1, Pad1, SMO, SpEH, and GoSCR) was constructed for converting L-PA **1** into 2-HAP **6**. Also, three expression plasmids (pETDuet-1, pRSFDuet-1, and pCDFDuet-1) were used for co-expression purposes of two different enzymes with various combinations (Additional file 1: Figure S1–S3). Theoretically, there are 15 different plasmid combinations, eight of them were randomly selected and constructed to co-express six enzymes. Specifically, three recombinant *E. coli* cells, *E. coli* (RFP-DEA-CGS), *E. coli* (DFP-CAS-REG), *E. coli* (CFP-DES-RGA) with three different plasmids combinations were successfully constructed (Fig. 1A). Furthermore, a SDS-PAGE analysis of the crude lysate present in the constructed recombinant *E. coli* cells showed that the six enzymes were successfully expressed in the *E. coli* cells (Fig. 1B). The performances of the constructed recombinant *E. coli* cells in the conversion of L-PA **1** into 2-HAP **6** were tested using 10 mM L-PA. The results showed that the recombinant *E. coli* (RFP-DEA-CGS) cells had the highest activity (5.6 U/g cdw) with the amino acid L-PA **1**. By using the recombinant *E. coli* (RFP-DEA-CGS) cells, the conversion percentage of 2-HAP **6** raised to 93% within a 7-h period (Fig. 1C). Then, a recombinant *E. coli* (RFP-DEA-CGS) was selected as the best candidate to convert L-PA **1** into 2-HAP **6**. The cell growth and specific activity of the recombinant *E. coli* (RFP-DEA-CGS) were further analyzed (Fig. 1D). Moreover, the highest activity value of *E. coli* (RFP-DEA-CGS) was 5.8 U/g cdw at OD_600_ (3.0) after a 14-h induction period.

### Conversion of L-PA 1 into 2-HAP 6 by *E. coli* (RFP-DEA-CGS)

**Fig. 2:**
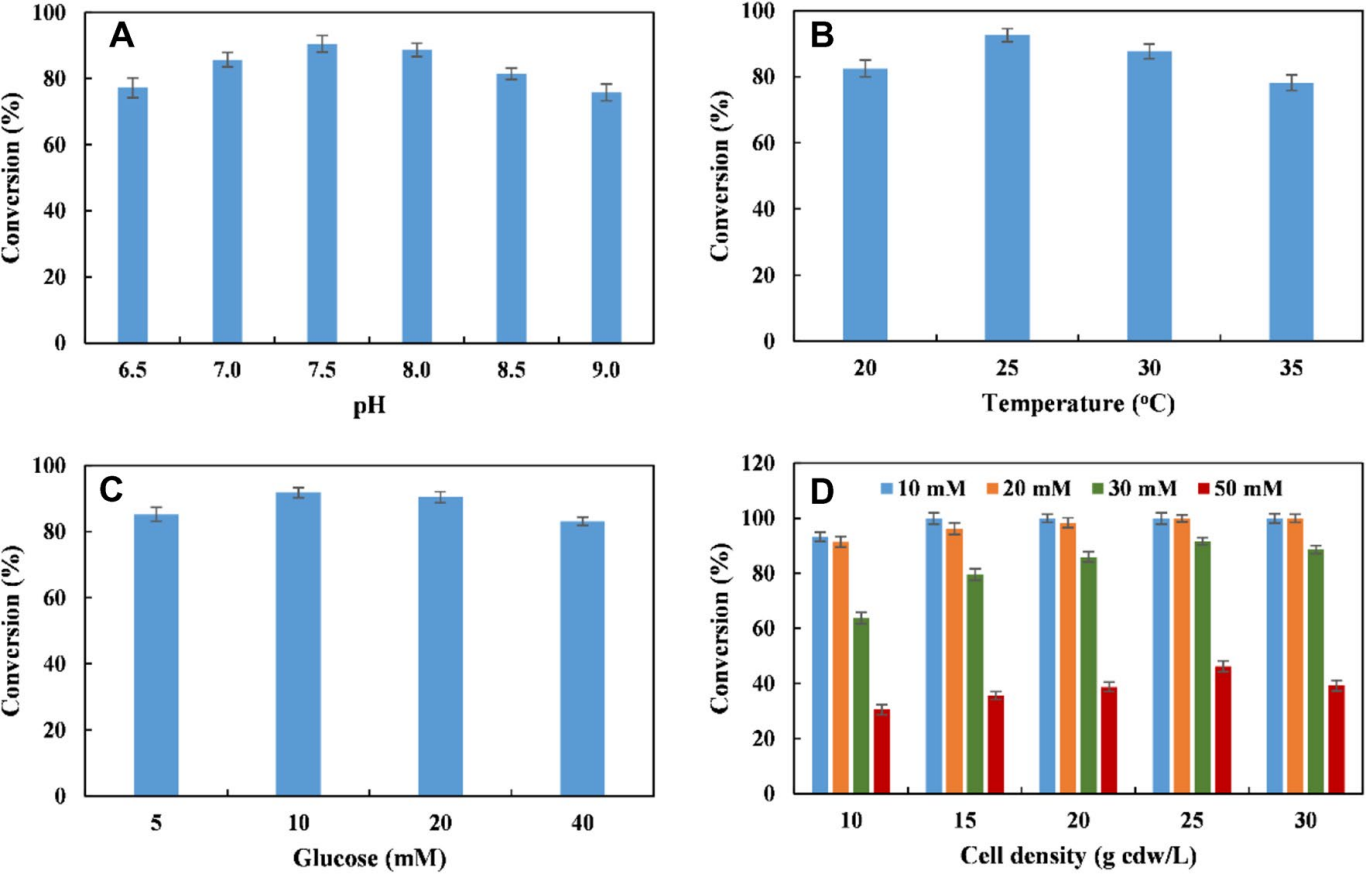
Reaction condition optimization for conversion of L-PA 1 to 2-HAP 6. **A** Effect of different pH on the conversion of 2-HAP **6**; **B** effect of different temperature on the conversion of 2-HAP **6**; **C** effect of glucose concentration on the conversion of 2-HAP **6**; **D** effect of different cell density of *E. coli* (RFP-DEA-CGS) on the conversion of different concentration of L-PA **6**, blue: 10 mM of L-PA; yellow: 20 mM of L-PA; green: 30 mM of L-PA; red: 50 mM of L-PA

After the recombinant *E. coli* (RFP-DEA-CGS) was selected for converting L-PA into 2-HAP, the reaction conditions were optimized with 20 mM L-PA. The highest conversion percentage of 2-HAP was 92% at pH 7.5 and 25 °C (Fig. 2A-B). Moreover, the addition of 10 mM glucose increased the conversion percentage of 2-HAP **6** to 95% (Fig. 2C). For 10 mM L-PA **1**, 15 g cdw/L of *E. coli* (RFP-DEA-CGS) was enough to convert L-PA **1** into 2-HAP **6** with a 99% conversion percentage. However, 25 g cdw/L of *E. coli* (RFP-DEA-CGS) was needed for conversion of 20 mM L-PA **1** into 2-HAP **6**. In the case of 30 mM L-PA, the conversion percentage of 2-HAP **6** was 91.6% using 25 g cdw/L of *E. coli* (RFP-DEA-CGS). However, the conversion of 2-HAP **6** for 50 mM L-PA decreased to 46.2% using 25 g cdw/L of *E. coli* (RFP-DEA-CGS) (Fig. 2D and Additional file 1: Figure S4). In fact, the 2-HAP **6** compound is a very important structural moiety in several synthetic intermediates used in organic and medicinal chemistry. For example, 2-HAP can be used as a building block in the synthesis of oxazolone carboxamides, which belong to a novel class of acid ceramidase inhibitors. Also, 2-HAP can act as a useful pharmacological tool in treating sphingolipid-mediated disorders (Caputo et al. 2020) and as a potent inhibitor of urease (Tanaka et al. 2004).

### Construction of recombinant *E. coli* cell modules for conversion of 2-HAP into chiral phenylglycinol

**Fig. 3:**
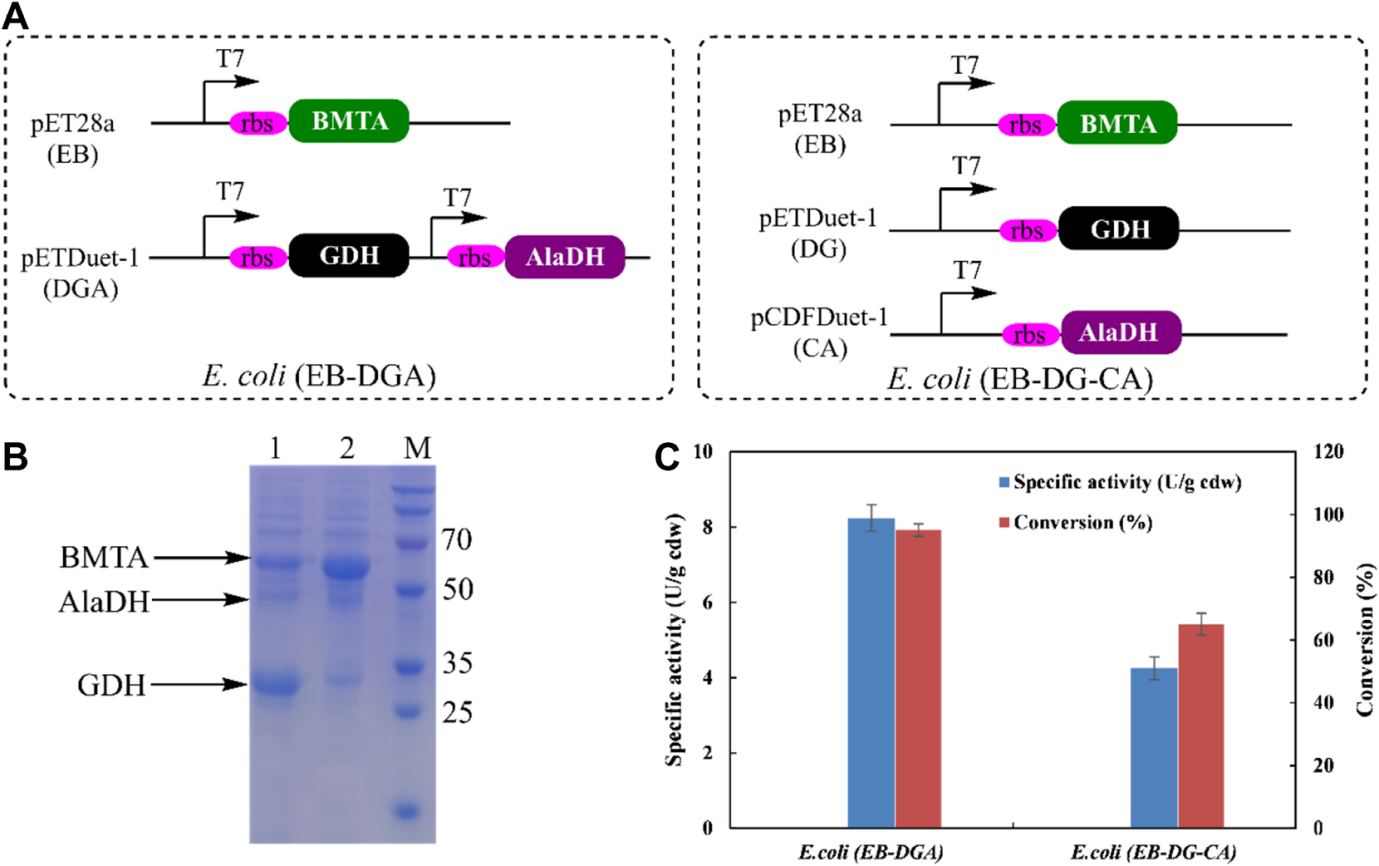
Construction of recombinant *E. coli* cells for conversion of 2-HAP 6 to (*R*)-phenylglycinol **7**. **A** Construction of recombinant *E. coli* cells co-expression of three enzymes (BMTA, AlaDH and GDH) based on different plasmid configurations. **B** SDS-PAGE analysis of recombinant *E. coli* cells. M: marker; lane 1: cell-free extract of *E. coli* (EB-DGA), lane 2: cell-free extract of *E. coli* (EB-DG-CA). **C** Activities of two different recombinant *E. coli* cells for conversion of 2-HAP **6** to (*R*)-phenylglycinol **7**

The MVTA enzyme was selected to catalyze the asymmetric amination of 2-HAP **6** into (*S*)-**7**, and R-MBA was used as an optimum amine donor in the conversion process to acetophenone (AP) (Zhang et al. 2019a). Therefore, the recombinant *E. coli* (MVTA) was then constructed for conversion of 2-HAP **6** into (*S*)-phenylglycinol **7**. In this case, the whole-cell specific activity of the recombinant *E. coli* (MVTA) cells with 2-HAP **6** was 22.2 U/g cdw. Regarding the bioconversion of 2-HAP **6** into (*R*)-**7**, BMTA was used for catalyzing the amination of the 2-HAP **6** into (*R*)-**7**. In addition, L-Ala serves as an optimum amine donor that can lead to pyruvate. Thus, pyruvate can then be recycled to alanine by employing the AlaDH enzyme, which consumes ammonia and NADH. Also, NADH can be recycled using GDH. Consequently, some recombinant *E. coli* cells were constructed to co-express BMTA, AlaDH, and GDH (Fig. 3A). In fact, an SDS-PAGE showed that all the enzymes were successfully expressed (Fig. 3B), and the activities of the recombinant *E. coli* cells were tested with 10 mM 2-HAP **6**. Therefore, the recombinant *E. coli* (EB-DGA) cells showed a highest activity of 8.3 U/g cdw with 2-HAP **6** and a 95% conversion percentage of (*R*)-**7** during a 12-h reaction period (Fig. 3C).

### Conversion of L-PA 1 into (*S*)-7 by the designed *E. coli* (RFP-DEA-CGS) and the *E. coli* (MVTA) consortium

**Fig. 4:**
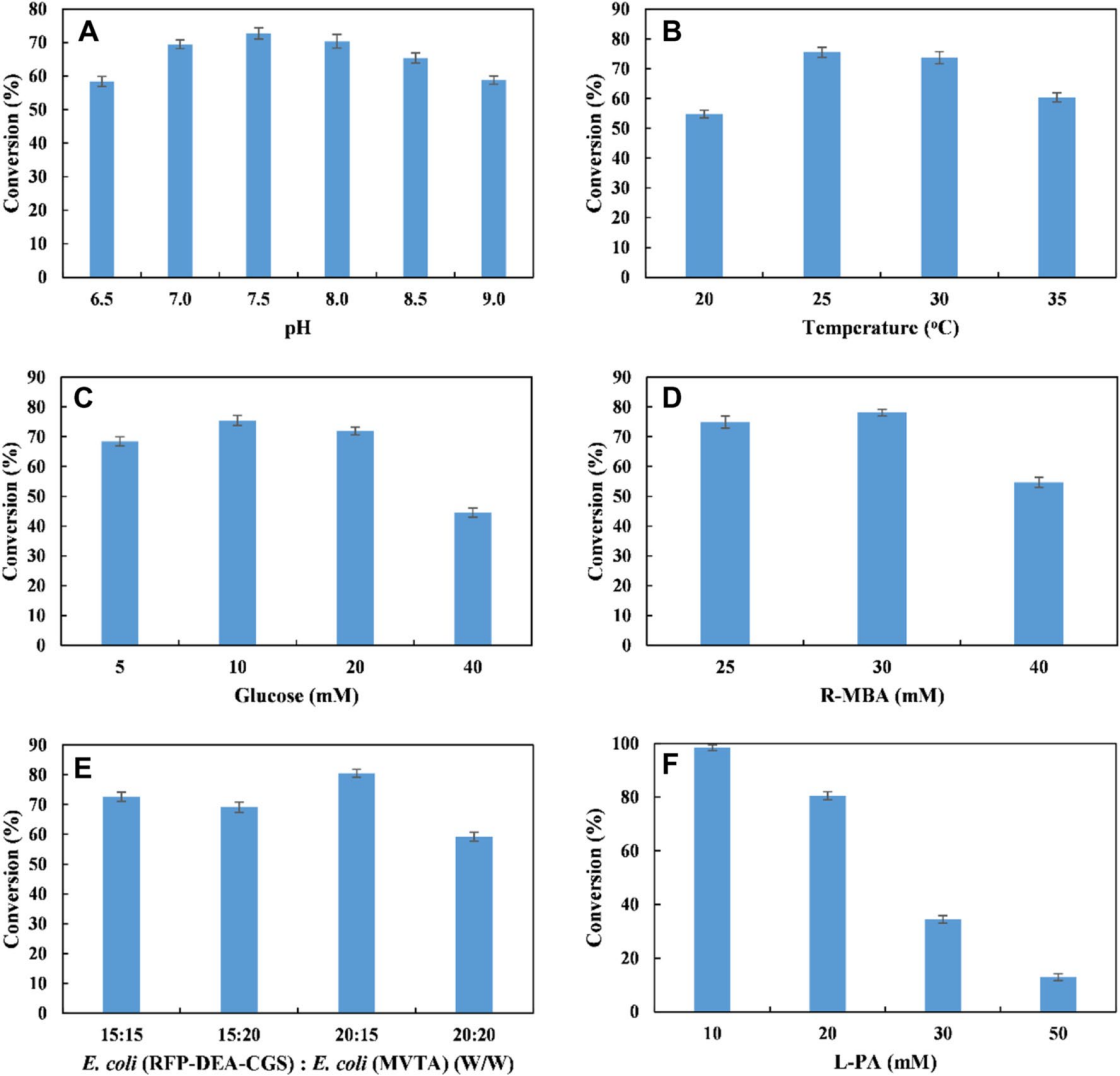
Reaction condition optimization for conversion of L-PA 1 (20 mM) to (*S*)-7 by the designed *E. coli* (RFP-DEA-CGS) and *E. coli* (MVTA) consortium. **A** Effect of different pH on the conversion of (*S*)-**7**; **B** effect of different temperature on the conversion of (*S*)-**7**; **C** effect of glucose concentration on the conversion of (*S*)-**7**; **D** effect of R-MBA concentration on the conversion of (*S*)-**7**; **E** effect of different cell density of *E. coli* (RFP-DEA-CGS) and *E. coli* (MVTA) on the conversion of (*S*)-**7**; **F** effect of different L-PA **1** concentration on the conversion of (*S*)-**7**

After the biocatalytic modules were constructed, the lyophilized *E. coli* (RFP-DEA-CGS) and *E. coli* (MVTA) cells were combined to convert L-PA **1** into (*S*)-**7**. The reaction conditions were first optimized with 20 mM of L-PA **1**. The results showed that the highest conversion percentage of (*S*)-**7** (78%) was obtained at a pH of 7.5 and 25 °C. In this case, 10 mM glucose and 30 mM R-MBA were used as substrates, and *E. coli* (RFP-DEA-CGS) and *E. coli* (MVTA) were combined at a 1:1 ratio with 15 g cdw/L of each recombinant (Fig. 4A–E). Moreover, the combination of two recombinant *E. coli* cells at a 2:1.5 ratio (20 g cdw/L of *E. coli* (RFP-DEA-CGS) and 15 g cdw/L of *E. coli* (MVTA)) resulted in the highest conversion of (*S*)-**7** (80%) and > 99% *ee*. Following the above optimized reaction conditions, different L-PA 1 concentrations (10–50 mM) were tested in a period of 7 h (Fig. 4F), and about 98% of conversion of (*S*)-7 was obtained with 10 mM L-PA **1**. Moreover, (*S*)-**7** was obtained with only a 34.4% conversion percentage for 30 mM L-PA **1**. Surprisingly, a further increase in the substrate concentration to 50 mM decreased the conversion percentage of (*S*)-**7** to 12.9%. In fact, the remaining intermediate product was mainly diol **5** (85%), and almost no 2-HAP **6** was detected in the reaction mixture. This finding implied that the reactivity of GoSCR with respect to the diol intermediate compound in this cascade biocatalysis system could be severely inhibited by R-MBA. Therefore, the effect of different concentrations of R-MBA (0–60 mM) on the conversion of 2-HAP **6** by *E. coli* (RFP-DEA-CGS) was examined with 20 mM L-PA **1**. The results showed that the conversion of 2-HAP **6** was significantly decreased with the increasing concentration of R-MBA (Additional file 1: Figure S5).

**Fig. 5:**
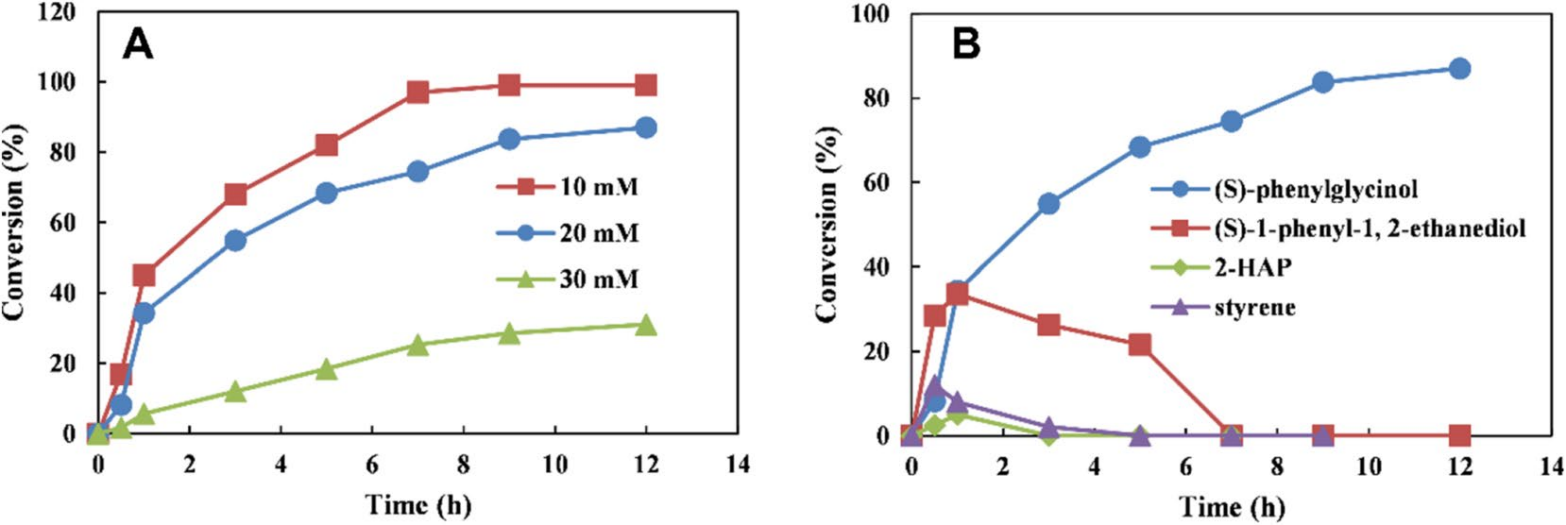
Time course of the cascade reaction for the synthesis of (*S*)-7 from L-PA 1. **A** Time course of the cascade reaction for conversion of 10–30 mM L-PA **1** to (*S*)-**7**; **B** time course of the cascade reaction for intermediate produced from L-PA **1**. Reaction conditions (5 mL): 100 mM phosphate buffer (pH 7.5), 10–30 mM L-PA, 10 mM glucose, 20 g cdw/L *E. coli* (RFP-DEA-CGS), 15 g cdw/L *E. coli* (MVTA), 30 mM (*R*)-MBA and 0.1 mM PLP, at 25 °C. All biotransformations were performed in duplicate and conversion was determined by GC, error limit: < 2% of the state values

The time course in the bioconversion of 10–30 mM L-PA **1** into (*S*)-**7** is shown in Fig. 5. In fact, (*S*)-**7** could be obtained with 99% and 84% conversion percentages (> 99% *ee*) from 10 and 20 mM L-PA at 12 h, respectively (Additional file 1: Figures S6 and S8). For 30 mM L-PA, the highest conversion percentage of (*S*)-**7** was only 35% (Fig. 5A). Furthermore, the analysis of the intermediate products produced from L-PA (20 mM) showed that the (*S*)-**7** (35%), diol **5** (35%), styrene **3** (8%), and 2-HAP **6** (5%) were the main remaining products in the reaction system at 1 h. This finding implied that 83% of L-PA **1** was converted to styrene **3** due to the high activity of PAL, Fdc1, and Pad1. After 1 h, the concentration of diol **5** began to decrease, while the concentration of (*S*)-**7** increased linearly. At 7 h, a trace amount of diol **5** (< 1%) and 2-HAP **6** (< 1%) were detected in the reaction mixture, and the conversion of (*S*)-**7** reached 75%. Finally, the conversion of (*S*)-**7** was continually increased to 84% at 12 h (Fig. 5B).

### Conversion of L-PA 1 into (*R*)-7 by the designed *E. coli* (RFP-DEA-CGS) and the *E. coli* (EB-DGA) consortium

**Fig. 6:**
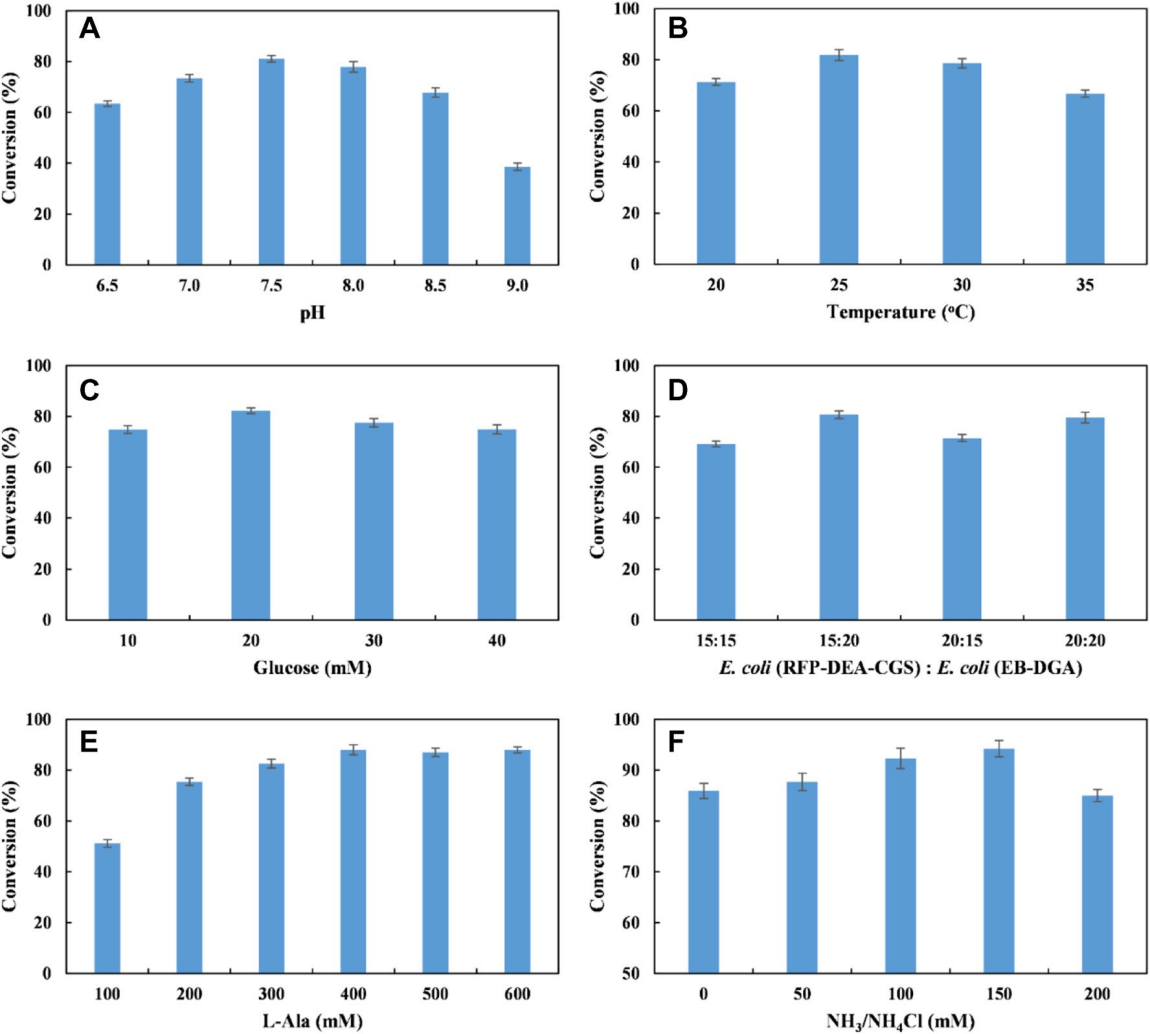
Reaction condition optimization for conversion of L-PA 1 (20 mM) to (*R*)-7 by the designed *E. coli* (RFP-DEA-CGS) and *E. coli* (EB-DGA) consortium. **A** Effect of different pH on the conversion of (*R*)-**7**; **B** effect of different temperature on the conversion of (*R*)-**7**; **C** effect of glucose concentration on the conversion of (*R*)-**7**; **D** effect of different cell density of *E. coli* (RFP-DEA-CGS) and *E. coli* (MVTA) on the conversion of (*R*)-**7**; **E** effect of L-Ala concentration on the conversion of (*R*)-**7**; F: effect of NH_3_/NH_4_Cl concentration on the conversion of (*R*)-**7**

**Fig. 7:**
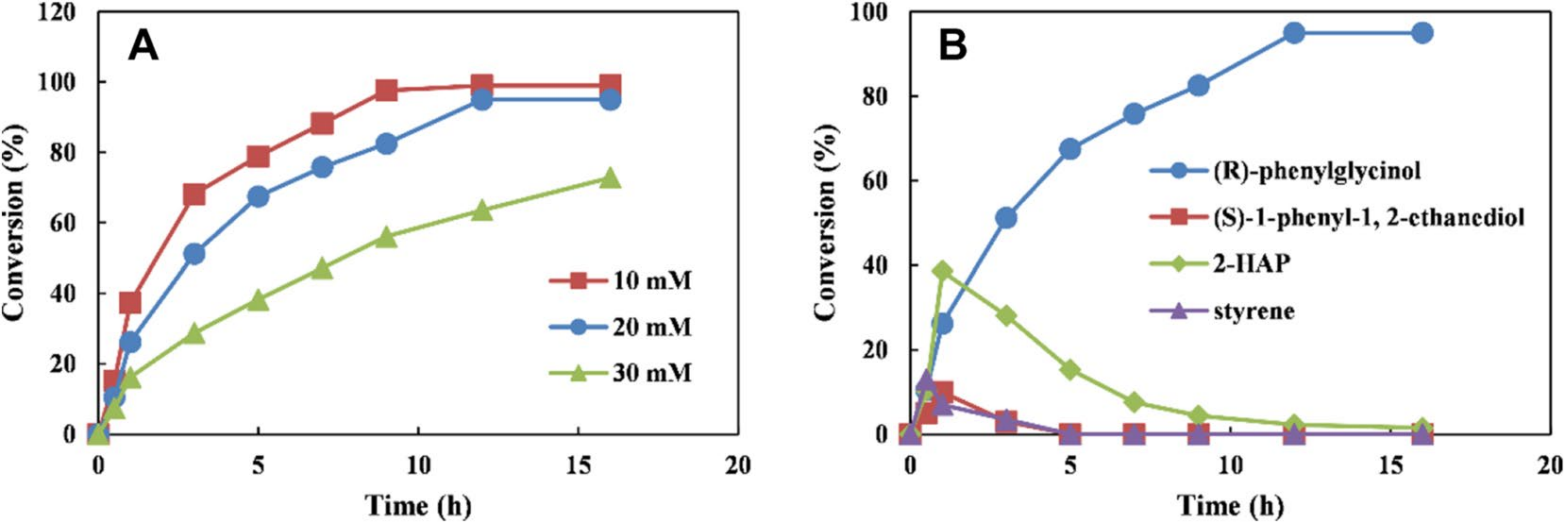
Time course of the cascade reaction for the synthesis of (*R*)-7 from L-PA 1. **A** Time course of the cascade reaction for conversion of 10–30 mM L-PA to (*R*)-**7**; **B** time course of the cascade reaction for intermediate produced from L-PA **1**. Reaction conditions (5 mL): 100 mM phosphate buffer (pH 7.5), 10–30 mM L-PA, 20 mM glucose, 15 g cdw/L *E. coli* (RFP-DEA-CGS), 20 g cdw/L *E. coli* (EB-DGA), 400 mM L-ala, 150 mM NH_3_/NH_4_Cl, 0.1 mM PLP, at 25 °C. All biotransformations were performed in duplicate and conversion was determined by GC, error limit: < 2% of the state values

The lyophilized *E. coli* (RFP-DEA-CGS) and *E. coli* (EB-DGA) cells were combined to create another *E. coli* microbial consortium for conversion purposes from L-PA **1** to (*R*)-**7**. The reaction conditions were optimized with 20 mM of L-PA **1**. Figure 6A–F shows that the combination of *E. coli* (RFP-DEA-CGS) and *E. coli* (EB-DGA) at a 1.5:2 ratio (15 g cdw/L of *E. coli* (RFP-DEA-CGS) and 20 g cdw/L of *E. coli* (EB-DGA) led to the highest conversion percentage of (*R*)-**7** (85%) at pH 7.5 and 25 °C with 20 mM glucose and 400 mM L-Ala. In addition, a reaction mixture with 150 mM NH_3_/NH_4_Cl (regeneration of L-Ala by AlaDH) resulted in a 95% conversion percentage of (*R*)-**7**. Further attempts to decrease the usage of L-Ala showed that the (*R*)-7 yield was not evidently improved (< 85%) when the L-Ala concentration was below 400 mM (data not shown). In fact, Koszelewski et al. (2010) reported that although only a catalytic amount of alanine is technically required in the L-Ala regeneration system, the non-favored reaction equilibrium of the amination step can delay the reaction. However, the excess of alanine allows a reasonable overall reaction rate. The time course for the conversion of 10–30 mM L-PA **1** into (*R*)-**7** is shown in Fig. 7. In fact, (*R*)**-7** was obtained with conversion percentages of 99% and 95% (> 99% *ee*) from 10 and 20 mM L-PA **1** at 12 h, respectively (Additional file 1: Figures S7–S8). However, (*R*)**-7** was obtained with a 72.8% conversion percentage and *ee* values higher than 99% using a 30-mM L-PA substrate after a 16-h reaction period (Fig. 7A). Specifically, an analysis of the intermediate products from the L-PA **1** (20 mM) reaction showed that 2-HAP **6** (38.6%), (*R*)-**7** (26.2%), diol **5** (10.0%), and styrene **3** (7%) were the main products obtained after 1 h. Therefore, L-PA **1** (> 84%) was converted into styrene **3** due to the high activity of PAL, Fdc1, and Pad1. In fact, this event is consistent with the conversion reaction of L-PA **1** into (*S*)**-7**. However, 2-HAP** 6** (38.6%) and diol **5** (10.0%) remained in this reaction system, which implies that most of the diol **5** compound was converted into 2-HAP **6**. Also, the activity of GoSCR was not affected in this system**.** After a 1-h reaction period, the concentration of 2-HAP began to decrease, while the concentration of (*S*)-**7** increased linearly. In fact, (*R*)-**7** was produced in about a 95% conversion percentage and > 99% *ee* at 12 h. Moreover, a trace of 2-HAP **6** (1.5%) was detected in the system, and the diol **5** and styrene **3** could not be detected at this reaction time (Fig. 7B).

### Preparation of (*S*)-7 and (*R*)-7 from L-PA

To examine the scalability of this cascade biocatalysis, preparative experiments were conducted on a 100-mL scale via the constructed recombinant *E. coli* microbial consortium. After the reactions were completed (12 h), the reaction solution was extracted with ethyl acetate and flash chromatography, pure (*S*)-**7** and (*R*)-**7** were prepared with 71.0% and 80.5% isolated yields, > 99% *ee*, and 5.19 g/L.d and 4.42 g/L.d volumetric productivity, respectively (Table 2, Additional file 1: Figures S9-S10).

**Table 2:** Preparation of (*S*)-7 and (*R*)-7 from L-PA 1 with the combined resting cell of *E. coli* (RFP-DEA-CGS) and *E. coli* (MVTA) or *E. coli* (EB-DGA) ^a^

Entry	L-PA 1 (mg)	Time (h)	7 (mg)	Yield of 7 (%)	*ee* of 7 (%)^b^	S.T.Y. (g/L d)
1	330.4	9	194.8	71.0	> 99(*S*)	5.19
2	330.4	12	220.9	80.5	> 99(*R*)	4.42

S.T.Y.: space–time yield

^a^ The reactions were conducted in 100 mL sodium phosphate buffer (100 mM, pH 7.5). The reactions containing 2 mmol (330.4 mg) substrates, 20 g cdw/L *E. coli* (RFP-DEA-CGS) and 15 g cdw/L *E. coli* (MVTA) or 15 g cdw/L *E. coli* (RFP-DEA-CGS) and 20 g cdw/L *E. coli* (EB-DGA), 10–20 mM glucose, 0.1 mM PLP, 30 mM (*R*)-MBA or 400 mM L-Ala and 150 mM NH_3_/NH_4_Cl, at 25 °C

^b^ *ee* was determined by chiral GC

## Conclusions

In general, an artificial biocatalytic cascade was successfully constructed in this study by using a recombinant *E. coli* microbial consortium for converting bio-based l-phenylalanine into chiral phenylglycinol. Seven kinds of enzymes (PAL, Pad1, Fdc1, SMO, SpEH, GoSCR, and MVTA or BMTA) were first examined for the in vitro conversion of these two compounds. Both (*R*)-phenylglycinol and (*S*)-phenylglycinol were obtained within a conversion percentage range of 66.8–87.2% (> 99% *ee*) from 10–20 mM l-phenylalanine. Additionally, a recombinant *E. coli* microbial consortium, that included two recombinant *E. coli* cell modules, was constructed for the efficient conversion of l-phenylalanine into chiral phenylglycinol (up to 99% and > 99% *ee*). Moreover, the synthetic potential of this cascade biocatalysis was further confirmed by the constructed recombinant *E. coli* microbial consortium [*E. coli* (RFP-DEA-CGS), *E. coli* (MVTA) or *E. coli* (RFP-DEA-CGS), *E. coli* (EB-DGA)]. In this case, (*S*)-phenylglycinol and (*R*)-phenylglycinol were obtained with good yields (71.0% and 80.5%, respectively) and *ee* values higher than 99%. Finally, the advantages of this method are its mild reaction conditions, good yields and excellent *ee* values, and not requiring additional cofactors. Therefore, this system can enhance the sustainable synthesis of (*S*)-phenylglycinol or (*R*)-phenylglycinol from bio-based L-phenylalanine.

### Supplementary Information

**Additional file 1: Table S1.** Primers used in this study. **Table S2.** Recombinant strains constructed in this study. **Table S3.** The specific activity of enzymes used in this study. **Figure S1.** Recombinant plasmid constructed. **Figure S2.** Recombinant *E. coli* cells co-expression of multiple enzymes. **Figure S3.** SDS-PAGE of the cell-free extracts of recombinant *E. coli* cells co-expression of multiple enzymes. A: lane M:marker, lane 1, 2: *E. coli* (DEA), lane 3, 4: *E. coli* (CGS); B: laneM:marker, lane 1, 2: *E. coli* (REG); C: laneM:marker, lane 1, 2: *E. coli* (DFP); D: laneM:marker, lane1: *E. coli* (CFP); E: laneM:marker, lane 1, 2: *E. coli* (RFP), lane 3: *E. coli* (DES); F: lane M:marker, lane 1, 2: *E. coli* (RGA); G: lane M:marker, lane 1: *E. coli* (CAS). **Figure S4.** Achiral GC chromatograms of 2-HAP. A: 2-HAP standard. B: 2-HAP produced by conversion of L-phenylalanine (10 mM) with resting cells of *E. coli* (RFP-DEA-CGS) (15 g cdw/L) at 3 h. C: 2-HAP produced by conversion of L-phenylalanine (20 mM) with resting cells of *E. coli* (RFP-DEA-CGS) (25 g cdw/L) at 9 h. D: 2-HAP produced by conversion of L-phenylalanine (50 mM) with resting cells of *E. coli* (RFP-DEA-CGS) (25 g cdw/L) at 12 h. IS: Internal standard (n-dodecane), 4.05 min; 2-HAP: 2-hydroxyacetophenone, 4.91 min. **Figure S5.** Effect of different concentration of RMBA on *E. coli* (RFP-DEA-CGS) cells for conversion of L-PA to 2-HAP. **Figure S6.** Achiral GC chromatograms of (*S*)-phenylglycinol. A: (*S*)-phenylglycinol standard. B: (S)-phenylglycinol produced by conversion of L-phenylalanine (10 mM) with resting cells of *E. coli* (RFP-DEA-CGS) (20 g cdw/L) and *E. coli* (MVTA) (15 g cdw/L) at 12 h. C: (*S*)-phenylglycinol produced by conversion of L-phenylalanine (20 mM) with resting cells of *E. coli* (RFPDEA-CGS) (20 g cdw/L) and *E. coli* (MVTA) (15 g cdw/L) at 12 h. IS: Internal standard (n-dodecane), 4.05 min; RMBA: (*R*)-(+)-1-phenylethylamine, 2.62 min; AP: acetophenone, 2.78 min; Product (S)-phenylglycinol, 6.05 min. **Figure S7.** Achiral GC chromatograms of (*R*)-phenylglycinol. A: (R)-phenylglycinol standard. B: (*R*)-phenylglycinol produced by conversion of L-phenylalanine (10 mM) with resting cells of *E. coli* (RFP-DEA-CGS) (15 g cdw/L) and *E. coli* (EB-DGA) (20 g cdw/L) at 9 h. C: (*R*)-phenylglycinol produced by conversion of L-phenylalanine (20 mM) with resting cells of *E. coli* (RFP-DEA-CGS) (15 g cdw/L) and *E. coli* (EB-DGA) (20 g cdw/L) at 12 h. IS: Internal standard (n-dodecane), 4.05 min; (R)-phenylglycinol, 6.05 min. **Figure S8.** Chiral GC chromatograms of phenylglycinol. A: (±)- phenylglycinol standard. B: (*R*)-phenylglycinol standard. C: (S)-phenylglycinol standard. D: (*R*)-phenylglycinol produced by conversion of L-phenylalanine (20 mM) with resting cells of **E . coli** (RFP-DEA-CGS) (15 g cdw/L) and *E. coli* (EB-DGA) (20 g cdw/L) at 12 h. E: (S)-phenylglycinol produced by conversion of L-phenylalanine (20 mM) with resting cells of *E. coli* (RFP-DEA-CGS) (20 g cdw/L) and *E. coli* (MVTA) (15 g cdw/L) at 9 h. (*R*)-phenylglycinol, 28.7 min; (S)-phenylglycinol, 29.3 min. **Figure S9.** Chiral GC chromatograms of phenylglycinol. A: (±)- phenylglycinol standard. B: (R)-phenylglycinol standard. C: (*S*)-phenylglycinol standard. D: Preparation of (*R*)-phenylglycinol from L-phenylalanine. E: Preparation of (S)-phenylglycinol from L-phenylalanine. (*R*)-phenylglycinol, 28.7 min; (*S*)-phenylglycinol, 29.3 min. **Figure S10.** ^1^H NMR spectra analysis of 7.

## Data Availability

All data generated or analyzed during this study are included in this article.

## Abbreviations

L-PA: l-Phenylalanine
2-HAP: 2-Hydroxyacetophenone
PAK4: P21-activated kinases
HDAC: Histone deacetylase
PDK1: 3-Phosphoinositide-dependent protein kinase-1
AA: Asymmetric aminohydroxylation
PAL: Phenylalanine ammonia lyase
PAD: Phenylacrylic acid decarboxylase
SMO: Styrene monooxygenase
EH: Epoxide hydrolase
ADH: Alcohol dehydrogenase
MVTA: (*R*)-Selective ω-transaminase from *Mycobacterium vanbaalenii*
BMTA: (*S*)-Selective ω-transaminase from *Bacillus megaterium* SC6394
AlaDH: Alanine dehydrogenase
GDH: Glucose dehydrogenase
R-MBA: (*R*)-α-Methylbenzylamine
PLP: Pyridoxal-5′-phosphate
IPTG: Isopropyl β-d-1-thiogalactopyranoside
LB: Luria–Bertani
TB: Terrific Broth
Fdc1: Ferulic acid decarboxylase
Pad1: Phenylacrylic acid decarboxylase
FID: Flame ionization detector
GoSCR: Polyol dehydrogenase from *Gluconobacter oxydans* 621H
EtOAc: Ethyl acetate
cdw: Cell dry weight
L-Ala: l-Alanine
