# Supplementary material for: One-pot synthesis of (R)- and (S)-phenylglycinol from bio-based l-phenylalanine by an artificial biocatalytic cascade
Source: Bioresour Bioprocess. 2021 Oct 6;8(1):97. doi: 10.1186/s40643-021-00448-5 (PMC10991228; doi:10.1186/s40643-021-00448-5)
Supplement: Supplementary file 1 — Additional file 1: Table S1. Primers used in this study. Table S2. Recombinant strains constructed in this study. Table S3. The specific activity of enzymes used in this study. Figure S1. Recombinant plasmid constructed. Figure S2. Recombinant E. coli cells co-expression of multiple enzymes. Figure S3. SDS-PAGE of the cell-free extracts of recombinant E. coli cells co-expression of multiple enzymes. A: lane M:marker, lane 1, 2: E. coli (DEA), lane 3, 4: E. coli (CGS); B: laneM:marker, lane 1, 2: E. coli (REG); C: laneM:marker, lane 1, 2: E. coli (DFP); D: laneM:marker, lane1: E. coli (CFP); E: laneM:marker, lane 1, 2: E. coli (RFP), lane 3: E. coli (DES); F: lane M:marker, lane 1, 2: E. coli (RGA); G: lane M:marker, lane 1: E. coli (CAS). Figure S4. Achiral GC chromatograms of 2-HAP. A: 2-HAP standard. B: 2-HAP produced by conversion of L-phenylalanine (10 mM) with resting cells of E. coli (RFP-DEA-CGS) (15 g cdw/L) at 3 h. C: 2-HAP produced by conversion of L-phenylalanine (20 mM) with resting cells of E. coli (RFP-DEA-CGS) (25 g cdw/L) at 9 h. D: 2-HAP produced by conversion of L-phenylalanine (50 mM) with resting cells of E. coli (RFP-DEA-CGS) (25 g cdw/L) at 12 h. IS: Internal standard (n-dodecane), 4.05 min; 2-HAP: 2-hydroxyacetophenone, 4.91 min. Figure S5. Effect of different concentration of RMBA on E. coli (RFP-DEA-CGS) cells for conversion of L-PA to 2-HAP. Figure S6. Achiral GC chromatograms of (S)-phenylglycinol. A: (S)-phenylglycinol standard. B: (S)-phenylglycinol produced by conversion of L-phenylalanine (10 mM) with resting cells of E. coli (RFP-DEA-CGS) (20 g cdw/L) and E. coli (MVTA) (15 g cdw/L) at 12 h. C: (S)-phenylglycinol produced by conversion of L-phenylalanine (20 mM) with resting cells of E. coli (RFPDEA-CGS) (20 g cdw/L) and E. coli (MVTA) (15 g cdw/L) at 12 h. IS: Internal standard (n-dodecane), 4.05 min; RMBA: (R)-(+)-1-phenylethylamine, 2.62 min; AP: acetophenone, 2.78 min; Product (S)-phenylglycinol, 6.05 min. Figure S7. Achiral GC ch [file 40643_2021_448_MOESM1_ESM.docx]

**Supplementary Material**

**One-pot synthesis of (*R*)- and (*S*)-phenylglycinol from bio-based L-phenylalanine by an artificial biocatalytic cascade**

Jiandong Zhang^1*^, Ning Qi^1^, Lili Gao^2^, Jing Li^1^, Chaofeng Zhang^1^, Honghong Chang^1^

^†^ Department of Biological and Pharmaceutical Engineering, College of Biomedical Engineering, Taiyuan University of Technology, Taiyuan, Shanxi, China

^‡^ [College of Environmental Science and Engineering](http://english.tyut.edu.cn/Schools___Departments/College_of_Environmental_Science_and_Engineering.htm), Taiyuan University of Technology, Taiyuan, Shanxi, China

**Corresponding author:*

*Phone: +86-351-6018534; Fax: +86-351-6018534*

*E-mail:* [*zhangjiandong@tyut.edu.cn*](mailto:zhangjiandong@tyut.edu.cn;) (J. D. Zhang)

Gene sequence of PAL：ATGGATCAAATCGAAGCAATGTTGTGCGGCGGAGGAGAGAAGACAAAAGTGGCGGTTACTACGAAGACTTTGGCAGATCCATTGAATTGGGGTTTAGCAGCGGATCAAATGAAAGGAAGTCATTTAGATGAAGTGAAGAAGATGGTCGAAGAGTATCGTAGACCAGTCGTGAATCTTGGCGGAGAAACACTGACGATCGGACAAGTTGCTGCCATCTCCACCGTAGGAGGCAGCGTTAAGGTTGAGTTAGCGGAGACTTCAAGAGCCGGTGTGAAAGCTAGCAGTGATTGGGTTATGGAGAGCATGAACAAAGGTACTGACAGTTACGGAGTCACCACCGGCTTTGGTGCTACTTCTCACCGGAGAACCAAAAACGGCACCGCATTACAAACAGAACTCATTAGATTTTTGAACGCCGGAATATTCGGAAACACGAAGGAGACATGTCACACACTGCCGCAATCCGCCACAAGAGCCGCCATGCTCGTCAGAGTCAACACTCTTCTCCAAGGATACTCCGGGATCCGATTCGAGATCCTCGAAGCGATTACAAGTCTCCTCAACCACAACATCTCTCCGTCACTACCTCTCCGTGGAACCATTACCGCCTCCGGCGATCTCGTTCCTCTCTCTTACATCGCCGGACTTCTCACCGGCCGTCCTAATTCCAAAGCCACCGGTCCCGACGGTGAATCGCTAACCGCGAAAGAAGCTTTTGAGAAAGCCGGAATCAGTACTGGATTCTTCGATTTACAACCTAAGGAAGGTTTAGCTCTCGTTAATGGCACGGCGGTTGGATCTGGAATGGCGTCGATGGTTCTATTCGAAGCGAATGTCCAAGCGGTGTTAGCGGAGGTTTTATCAGCGATCTTCGCGGAGGTTATGAGCGGGAAACCTGAGTTTACCGATCATCTGACTCATCGTTTAAAACATCATCCCGGACAAATCGAAGCGGCGGCGATAATGGAGCACATACTCGACGGAAGCTCATACATGAAATTAGCTCAAAAGGTTCACGAGATGGATCCATTGCAGAAACCAAAACAAGATCGTTACGCTCTTCGTACATCTCCTCAATGGCTAGGTCCTCAAATTGAAGTAATCCGTCAAGCTACGAAATCGATAGAGCGTGAAATCAACTCCGTTAACGATAATCCGTTGATCGATGTTTCGAGGAACAAGGCGATTCACGGTGGTAACTTCCAAGGAACACCAATCGGAGTTTCTATGGATAACACGAGATTGGCGATTGCTGCGATTGGGAAGCTAATGTTTGCTCAATTCTCTGAGCTTGTTAATGATTTCTACAACAATGGACTTCCTTCGAATCTAACTGCTTCGAGTAATCCAAGTTTGGATTATGGATTCAAAGGAGCAGAGATTGCTATGGCTTCTTATTGTTCTGAGCTTCAATACTTGGCTAATCCAGTCACAAGCCATGTTCAATCAGCTGAGCAACATAATCAAGATGTGAACTCTCTTGGTTTGATCTCGTCTCGTAAAACATCTGAAGCTGTGGATATTCTTAAGCTAATGTCAACAACGTTCCTTGTGGGGATATGTCAAGCTGTTGATTTGAGACATTTGGAGGAGAATCTGAGACAAACTGTGAAGAACACAGTTTCTCAAGTTGCTAAGAAAGTGTTAACCACTGGAATCAACGGTGAGTTACATCCGTCAAGGTTTTGCGAGAAGGACTTGCTTAAGGTTGTTGATCGTGAGCAAGTGTTCACGTATGTGGATGATCCTTGTAGCGCTACGTACCCGTTGATGCAGAGACTAAGACAAGTTATTGTTGATCACGCTTTGTCCAACGGTGAGACTGAGAAGAATGCAGTGACTTCGATCTTTCAAAAGATTGGAGCTTTTGAAGAGGAGCTTAAGGCTGTGCTTCCAAAGGAAGTTGAAGCGGCTAGAGCGGCTTATGGGAATGGAACTGCGCCGATTCCTAACCGGATTAAGGAATGTAGGTCGTATCCGTTGTATAGGTTCGTGAGGGAAGAGCTTGGAACGAAGTTGTTGACTGGAGAAAAGGTTGTGTCTCCGGGAGAGGAGTTTGATAAGGTCTTCACTGCTATGTGTGAAGGTAAACTTATTGATCCGTTGATGGATTGTCTCAAGGAATGGAACGGAGCTCCGATTCCGATTTGCTAA

Gene sequence of Fdc1:

ATGAGCGCGCAACCTGCGCACCTGTGCTTCCGCAGTTTCGTGGAAGCACTGAAAGTTGATAACGATCTGGTGGAAATTAATACCCCGATCGATCCGAACCTGGAAGCGGCGGCAATTACCCGTCGCGTGTGCGAAACGAATGATAAAGCCCCGCTGTTTAACAATCTGATTGGCATGAAAAACGGTCTGTTCCGCATCCTGGGTGCACCGGGCAGTCTGCGTAAAAGCTCTGCGGATCGTTATGGTCGTCTGGCACGTCATCTGGCACTGCCGCCGACCGCAAGCATGCGTGAAATTCTGGATAAAATGCTGAGTGCGAGCGATATGCCGCCGATTCCGCCGACCATCGTGCCGACGGGTCCGTGTAAAGAAAATAGCCTGGATGATTCTGAATTTGATCTGACCGAACTGCCGGTTCCGCTGATCCATAAAAGCGATGGCGGTAAATATATTCAGACGTACGGTATGCACATCGTGCAGAGTCCGGATGGCACCTGGACGAATTGGAGCATTGCGCGTGCGATGGTGCATGATAAAAACCACCTGACCGGTCTGGTGATCCCGCCGCAGCATATTTGGCAGATCCACCAGATGTGGAAAAAAGAAGGTCGTAGCGATGTTCCGTGGGCACTGGCATTCGGCGTGCCGCCGGCGGCAATTATGGCGAGTAGCATGCCGATCCCGGATGGTGTTACCGAAGCGGGTTATGTGGGCGCCATGACGGGCTCTAGTCTGGAACTGGTTAAATGCGATACCAACGATCTGTACGTTCCGGCGACGTCTGAAATTGTGCTGGAAGGCACCCTGTCTATCAGTGAAACGGGTCCGGAAGGCCCGTTTGGTGAAATGCATGGCTATATTTTCCCGGGTGATACCCACCTGGGCGCCAAATATAAAGTGAATCGCATTACGTACCGTAACAATGCAATCATGCCGATGAGCAGCTGCGGTCGCCTGACCGATGAAACCCATACGATGATTGGCAGCCTGGCAGCGGCCGAAATCCGTAAACTGTGTCAGCAGAACGATCTGCCGATCACGGATGCATTTGCGCCGTTCGAAAGCCAGGTGACCTGGGTTGCCCTGCGCGTTGATACGGAAAAACTGCGTGCAATGAAAACCACGTCTGAAGGTTTTCGTAAACGCGTGGGCGATGTGGTTTTCAATCATAAAGCGGGTTATACCATTCACCGCCTGGTGCTGGTTGGTGATGATATCGATGTTTACGAAGGCAAAGATGTGCTGTGGGCCTTTTCTACCCGTTGTCGCCCGGGTATGGATGAAACGCTGTTTGAAGATGTTCGCGGCTTCCCGCTGATTCCGTACATGGGTCATGGCAACGGTCCGGCACACCGTGGCGGTAAAGTTGTTAGTGATGCCCTGATGCCGACCGAATATACCACGGGTCGTAATTGGGAAGCAGCGGATTTTAACCAGTCTTACCCGGAAGACCTGAAACAGAAAGTGCTGGATAATTGGACCAAAATGGGCTTCAGTAACTAA

Gene sequence of Pad1:

ATGTTCAACTCACTTCTGTCCGGCACTACTACACCAAACTCCGGCCGTGCAAGCCCTCCGGCAAGCGAAATGCCGATTGATAACGACCATGTTGCAGTCGCACGTCCGGCACCGCGTCGCCGTCGCATCGTGGTTGCAATGACCGGTGCAACGGGTGCAATGCTGGGCATTAAAGTGCTGATCGCCCTGCGTCGCCTGAACGTCGAAACCCACCTGGTGATGAGTAAATGGGCAGAAGCTACCATTAAATATGAAACGGATTACCATCCGTCAAATGTGCGCGCGCTGGCCGATTATGTTCACAACATTAATGACATGGCGGCCCCGGTTAGCTCTGGCAGCTTTCGTGCGGATGGTATGATCGTCGTGCCGTGCTCTATGAAAACCCTGGCAGCTATTCATAGTGGCTTCTGTGATGACCTGATCTCCCGCACGGCAGATGTCATGCTGAAAGAACGTCGCCGTCTGGTGCTGGTTGCTCGTGAAACCCCGCTGTCCGAAATCCACCTGCGCAACATGCTGGAAGTTACGCGTGCAGGTGCTGTTATTTTTCCGCCGGTCCCGGCATTCTACATCAAAGCTGGCTCAATTGAAGATCTGATCGACCAGTCGGTGGGTCGCATGCTGGACCTGTTTGATCTGGACACCGGCGACTTCGAACGTTGGAATGGTTGGGAAAAATAA

Gene sequence of AlaDH:

ATGATCATAGGGGTTCCTAAAGAGATAAAAAACAATGAAAACCGTGTCGCATTAACACCCGGGGGCGTTTCTCAGCTCATTTCAAACGGCCACCGGGTGCTGGTTGAAACAGGCGCGGGCCTTGGAAGCGGATTTGAAAATGAAGCCTATGAGTCAGCAGGAGCGGAAATCATTGCTGATCCGAAGCAGGTCTGGGACGCCGAAATGGTCATGAAAGTAAAAGAACCGCTGCCGGAAGAATATGTTTATTTTCGCAAAGGACTTGTGCTGTTTACGTACCTTCATTTAGCAGCTGAGCCTGAGCTTGCACAGGCCTTGAAGGATAAAGGAGTAACTGCCATCGCATATGAAACGGTCAGTGAAGGCCGGACATTGCCTCTTCTGACGCCAATGTCAGAGGTTGCGGGCAGAATGGCAGCGCAAATCGGCGCTCAATTCTTAGAAAAGCCTAAAGGCGGAAAAGGCATTCTGCTTGCCGGGGTGCCTGGCGTTTCCCGCGGAAAAGTAACAATTATCGGAGGAGGCGTTGTCGGGACAAACGCGGCGAAAATGGCTGTCGGCCTCGGTGCAGATGTGACGATCATTGACTTAAACGCAGACCGCTTGCGCCAGCTTGATGACATCTTCGGCCATCAGATTAAAACGTTAATTTCTAATCCGGTCAATATTGCTGATGCTGTGGCGGAAGCGGATCTCCTCATTTGCGCGGTATTAATTCCGGGTGCTAAAGCTCCGACTCTTGTCACTGAGGAAATGGTAAAACAAATGAAACCCGGTTCAGTTATTGTTGATGTAGCGATCGACCAAGGCGGCATCGTCGAAACTGTCGACCATATCACAACACATGATCAGCCAACATATGAAAAACACGGGGTTGTGCATTATGCTGTAGCGAACATGCCAGGCGCAGTCCCTCGTACATCAACAATCGCCCTGACTAACGTTACTGTTCCATACGCGCTGCAAATCGCGAACAAAGGGGCAGTAAAAGCGCTCGCAGACAATACGGCACTGAGAGCGGGTTTAAACACCGCAAACGGACACGTGACCTATGAAGCTGTAGCAAGAGATCTAGGCTATGAGTATGTTCCTGCCGAGAAAGCTTTACAGGATGAATCATCTGTGGCGGGTGCTTAA

**Table S1.** Primers used in this study.

| Entry | Names | Primers(5’-3’) | REA^a^ |
| --- | --- | --- | --- |
| 1 | pET28a-PAL-*Nde*I-F | GGGAATTCCATATGGATCAAATCGAAGCAATGTTGTGCG | *Nde*I |
| 2 | pET28a-PAL-*Xho*I-R | CCGCTCGAGTTAGCAAATCGGAATCGGAGC | *Xho*I |
| 3 | pET28a-Fdc1-*Nde*I-F | GGGAATTCCATATGAGCGCGCAACCTGCG | *Nde*I |
| 4 | pET28a-Fdc1 -*Xho*I-R | CCGCTCGAGTTAGTTACTGAAGCC | *Xho*I |
| 5 | pET28a-Pad1-*Nde*I-F | GGGAATTCCATATGTTCAACTCACTTCTGTCCG | *Nde*I |
| 6 | pET28a-Pad1*-Xho*I-R | CCGCTCGAGTTATTTTTCCCAACCATTC | *Xho*I |
| 7 | pETduet-AlaDH-*BamH*I-F | CGCGGATCCGATGATCATAGGGGTTCCTAAAG | *BamH*I |
| 8 | pETduet-AlaDH-*PstI*-R | TGCACTGCAGTTAAGCACCCGCCACAG | *Pst*I |
| 9 | pCDFduet AlaDH-*BamH*I-F | CGCGGATCCGATGATCATAGGGGTTCCTAAAG | *BamH*I |
| 10 | pCDFduet AlaDH-*PstI*-R | TGCACTGCAGTTAAGCACCCGCCACAG | *Pst*I |
| 11 | pRSFduet-Fdc1-*BamH*I-F | CGCGGATCCGATGAGCGCGCAACCTGC | *BamH*I |
| 12 | pRSFduet-Fdc1-*Not*I-R | AAATATGCGGCCGCTTAGTTACTGAAGCCCATTTTGGTCC | *Not*I |
| 13 | pRSFduet-o-Pad1-*Nde*I-F | GGGAATTCCATATGTTCAACTCACTTCTGTCCG | *Nde*I |
| 14 | pRSFduet-o-Pad1-*Xho*I-R | CCGCTCGAGTTATTTTTCCCAACCATTC | *Xho*I |
| 15 | pETduet-SpEH-*BamH*I-F | CGCGGATCCGATGAACGTCGAACATATCCG | *BamH*I |
| 16 | pETduet-SpEH*-Hind*III-R | CCCAAGCTTTCAAAGATCCATCTGTGCAAAGGCC | *Hind*III |
| 17 | pETduet-o-PAL-*Nde*I-F | GGGAATTCCATATGGATCAAATCGAAGCAATGTTGTGCG | *Nde*I |
| 18 | pETduet-o-PAL-*Xho*I-R | CCGCTCGAGTTAGCAAATCGGAATCGGAGC | *Xho*I |
| 19 | pCDFduet-GoSCR-*BamH*I-F | CGCGGATCCGATGTACATGGAAAAACTCCGCC | *BamH*I |
| 20 | pCDFduet-GoSCR-*Not*I-R | AAATATGCGGCCGCTCACCAGACGGTGAAGC | *Not*I |
| 21 | pCDFduet-o-SMO(A)-*Nde*I-F | GGGAATTCCATATGAAAAAGCGTATCGGTATTGTTGGTGC | *Nde*I |
| 22 | pCDFduet-o-SMO(A)*-Kpn*I*-*R | CGGGGTACCTCAGGCCGCGATAGTGG | *Kpn*I |
| 23 | pCDFduet-o-o-SMO(B)-*Kpn*I-F | CGGGGTACCAAGGAGATATACCATGACGTTAAAAAAAG | *Kpn*I |
| 24 | pCDFduet-o-o-SMO(B)-*Xho*I-R | CCGCTCGAGTCAATTCAGTGGCAACGG | *Xho*I |
| 25 | pETduet -Fdc1-*BamH*I-F | CGCGGATCCGATGAGCGCGCAACCTGC | *BamH*I |
| 26 | pETduet-Fdc1-*Not*I-R | AAATATGCGGCCGCTTAGTTACTGAAGCCCATTTTGGTCC | *Not*I |
| 27 | pETduet-o-Pad1-*Nde*I-F | GGGAATTCCATATGTTCAACTCACTTCTGTCCG | *Nde*I |
| 28 | pETduet-o-Pad1-*Xho*I-R | CCGCTCGAGTTATTTTTCCCAACCATTC | *Xho*I |
| 29 | pCDFduet-PAL-*EcoR*I-F | CCGGAATTCGATGGATCAAATCGAAGCAATG | *EcoR*I |
| 30 | pCDFduet-PAL*-Not*I-R | AAATATGCGGCCGCTTAGCAAATCGGAATC | *Not*I |
| 31 | pCDFduet-o-SMO(A)-*Nde*I-F | GGGAATTCCATATGAAAAAGCGTATCGGTATTGTTGGTGC | *Nde*I |
| 32 | pCDFduet-o-SMO(A)*-Kpn*I*-*R | CGGGGTACCTCAGGCCGCGATAGTGG | *Kpn*I |
| 33 | pCDFduet-o-o-SMO(B)-KpnI-F | CGGGGTACCAAGGAGATATACCATGACGTTAAAAAAAG | *Kpn*I |
| 34 | pCDFduet-o-o-SMO(B)-*Xho*I-R | CCGCTCGAGTCAATTCAGTGGCAACGG | *Xho*I |
| 35 | pRSFduet-SpEH-*BamH*I-F | CGCGGATCCGATGAACGTCGAACATATCCG | *BamH*I |
| 36 | pRSFduet-SpEH*-Hind*III-R | CCCAAGCTTTCAAAGATCCATCTGTGCAAAGGCC | *Hind*III |
| 37 | pRSFduet-o-GoSCR-*Nde*I-F | GGGAATTCCATATGTACATGGAAAAACTCCGCC | *Nde*I |
|  |  |  |  |

**Table S1.** Continued

| 38 | pRSFduet-o-GoSCR-*Xho*I-R | CCGCTCGAGTCACCAGACGGTGAAGC | *Xho*I |
| --- | --- | --- | --- |
|  |  |  |  |
| 39 | pCDFduet-FDC-*BamH*I-F | CGCGGATCCGATGAGCGCGCAACCTGC | *BamH*I |
| 40 | pCDFduet-FDC-*Not*I-R | AAATATGCGGCCGCTTAGTTACTGAAGCCCATTTTGGTCC | *Not*I |
| 41 | pCDFduet-o-PAD-*Nde*I-F | GGGAATTCCATATGTTCAACTCACTTCTGTCCG | *Nde*I |
| 42 | pCDFduet-o-PAD-*Xho*I-R | CCGCTCGAGTTATTTTTCCCAACCATTC | *Xho*I |
| 43 | pETduet-SpEH-*BamH*I-F | CGCGGATCCGATGAACGTCGAACATATCCG | *BamH*I |
| 44 | pETduet-SpEH*-Hind*III-R | CCCAAGCTTTCAAAGATCCATCTGTGCAAAGGCC | *Hind*III |
| 45 | pETduet-o-SMO(A)-*Nde*I-F | GGGAATTCCATATGAAAAAGCGTATCGGTATTGTTGGTGC | *Nde*I |
| 46 | pETduet-o-SMO(A)*-Kpn*I*-*R | CGGGGTACCTCAGGCCGCGATAGTGG | *Kpn*I |
| 47 | pETduet-o-o-SMO(B)-*Kpn*I-F | CGGGGTACCAAGGAGATATACCATGACGTTAAAAAAAG | *Kpn*I |
| 48 | pETduet-o-o-SMO(B)-*Xho*I-R | CCGCTCGAGTCAATTCAGTGGCAACGG | *Xho*I |
| 49 | pRSFduet-GoSCR-*BamH*I-F | CGCGGATCCGATGTACATGGAAAAACTCCGCC | *BamH*I |
| 50 | pRSFduet-GoSCR-*Not*I-R | AAATATGCGGCCGCTCACCAGACGGTGAAGC | *Not*I |
| 51 | pRSFduet-o-PAL-*Nde*I-F | GGGAATTCCATATGGATCAAATCGAAGCAATGTTGTGCG | *Nde*I |
| 52 | pRSFduet-o-PAL-*Xho*I-R | CCGCTCGAGTTAGCAAATCGGAATCGGAGC | *Xho*I |
| 53 | pET28a-BMTA-*BamH*I-F | CGCGGATCCATGAGCCTGACGGTGCAGAAAATTAATTG | *BamH*I |
| 54 | pET28a-BMTA-*Xho*I-R | CCGCTCGAGTTACTGCCATTCACCACTCTCC | *Xho*I |
| 55 | pETduet-GDH-*BamH*I-F | CGCGGATCCGATGTATCCGGATTTAAAAGG | *BamH*I |
| 56 | pETduet-GDH-*Hind*III-R | CCCAAGCTTTTAACCGCGGCCTGC | *Hind*III |
| 57 | pETduet-o-AlaDH-*Kpn*I-F | CGGGGTACCATGATCATAGGGGTTCCTAAAG | *Kpn*I |
| 58 | pETduet-o-AlaDH*-Xho*I-R | CCGCTCGAGTTAAGCACCCGCCACAGATG | *Xho*I |
| 59 | pCDFduet-AlaDH-*Kpn*I-F | CGGGGTACCATGATCATAGGGGTTCCTAAAG | *Kpn*I |
| 60 | pCDFduet-AlaDH*-Xho*I-R | CCGCTCGAGTTAAGCACCCGCCACAGATG | *Xho*I |

^a^ REA: restriction endonuclease

**Table S2.** Recombinant strains constructed in this study.

| **Recombinant *E. coli*** | **Recombinant plasmid** | **Enzyme expressed** | **Reference** |
| --- | --- | --- | --- |
| *E. coli* (PAL) | pET28a-PAL | PAL (phenylalanine ammonia lyase) | This study |
| *E. coli* (FDC) | pET28a-FDC | FDC (ferulic acid decarboxylase) | This study |
| *E. coli* (PAD) | pET28a-PAD | PAD (phenylacrylic acid decarboxylase) | This study |
| *E. coli* (SMO) | pET28a-SMO | SMO ( styrene monooxygenase ) | [1] |
| *E. coli* (SpEH) | pETduet-SpEH | SpEH (epoxide hydrolase) | [2] |
| *E. coli* (GoSCR) | pET28a-GoSCR | GoSCR (alcohol dehydrogenase) | [3] |
| *E. coli* (MVTA) | pET28a-MVTA | MVTA (Transaminase) | [4] |
| *E. coli* (BMTA) | pET28a-BMTA | BMTA (Transaminase) | [5] |
| *E. coli* (AlaDH) | pETduet-AlaDH | AlaDH (alanine dehydrogenase ) | This study |
| *E. coli* (AlaDH) | pCDFduet-AlaDH | AlaDH (alanine dehydrogenase ) | This study |
| *E. coli* (GDH) | pETduet-GDH | GDH ( glucose dehydrogenase ) | [3] |
| *E. coli* (RFP) | pRSFduet-FDC-PAD | FDC and PAD | This study |
| *E. coli* (DEA) | pETduet-SpEH-PAL | SpEH and PAL | This study |
| *E. coli* (CGS) | pCDFduet-GoSCR-SMO | GoSCR and SMO | This study |
| *E. coli* (DFP) | pETduet-FDC-PAD | FDC and PAD | This study |
| *E. coli* (CAS) | pCDFduet-PAL-SMO | PAL and SMO | This study |
| *E. coli* (REG) | pRSFduet-SpEH-GoSCR | SpEH and GoSCR | This study |
| *E. coli* (CFP) | pCDFduet-FDC-PAD | FDC and PAD | This study |
| *E. coli* (DES) | pETduet-SpEH-SMO | SpEH and SMO | This study |
| *E. coli* (RGA) | pRSFduet-GoSCR-PAL | GoSCR and PAL | This study |
| *E. coli* (DGA) | pETduet-GDH-AlaDH | GDH and AlaDH | This study |
| *E. coli* (RFP-DEA-CGS) | pRSFduet-FDC-PAD  pETduet-SpEH-PAL  pCDFduet-GoSCR-SMO | PAL, FDC, PAD, SMO, SpEH and GOSCR | This study |
| *E. coli* (DFP-CAS-REG) | pETduet-FDC-PAD  pCDFduet-PAL-SMO  pRSFduet-SpEH-GoSCR | PAL, FDC, PAD, SMO, SpEH and GOSCR | This study |
| *E. coli* (CFP-DES-RGA) | pCDFduet-FDC-PAD  pETduet-SpEH-SMO  pRSFduet-GoSCR-PAL | PAL, FDC, PAD, SMO, SpEH and GOSCR | This study |
| *E. coli* (EB-DGA) | pET28a-BMTA  pETduet-GDH-AlaDH | BMTA, AlaDH and GDH | This study |
| *E. coli* (EB-DG-CA) | pET28a-BMTA  pETduet-GDH  pCDFduet-AlaDH | BMTA, AlaDH and GDH | This study |

**Table S3.** The specific activity of enzymes used in this study.

| **Enzyme** | **Specific activity (U/mg)** |
| --- | --- |
| PAL | 0.21 |
| Fdc1/Pad1 | 0.95 |
| SMO | 0.28 |
| SpEH | 0.48 |
| GoSCR | 0.14 |
| MVTA | 3.15 |
| BMTA | 0.97 |
| AlaDH | 1.20 |
| GDH | 3.14 |

**Figure S1**. Recombinant plasmid constructed.

**Figure S2**. Recombinant *E. coli* cells co-expression of multiple enzymes.


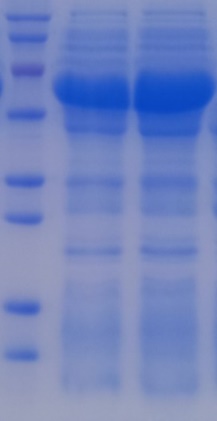


M 1 2

kDa

**FDC**

**PAD**

50

70

35

25

20

15


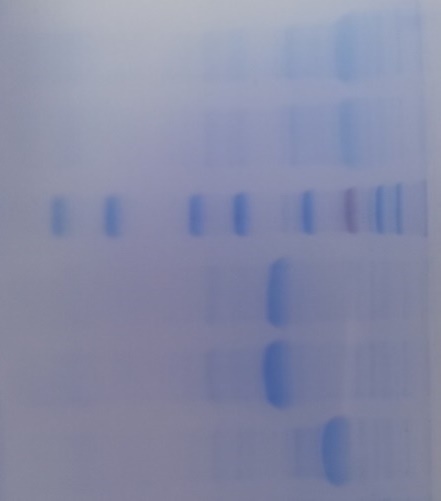


M 1 2

kDa

**SpEH**

**GoSCR**

50

70

35

25

20

15


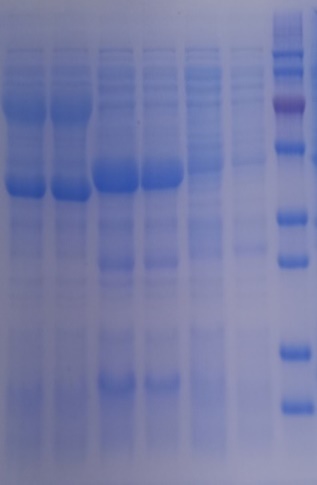

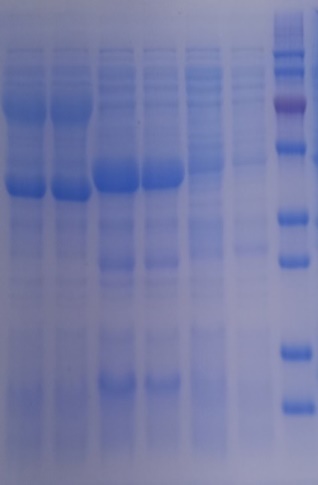


100

kDa

1 2 3 4 M

**PAL**

**SMO(styA)**

**SpEH**

**GoSCR**

**SMO(styB)**

70

50

35

25

20

15

C

A

B


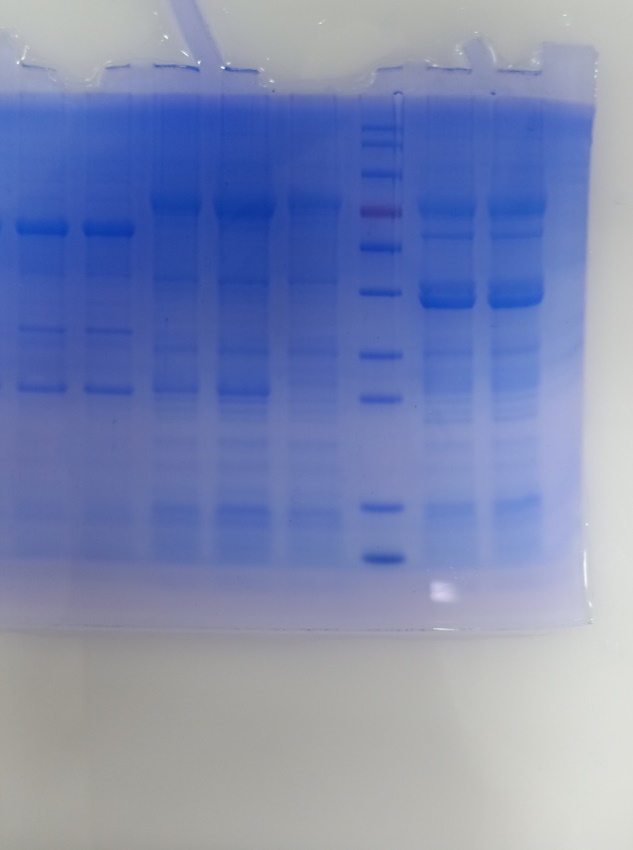


1 2 M

kDa

70

100

55

43

33

25

17

**PAL**

**GoSCR**


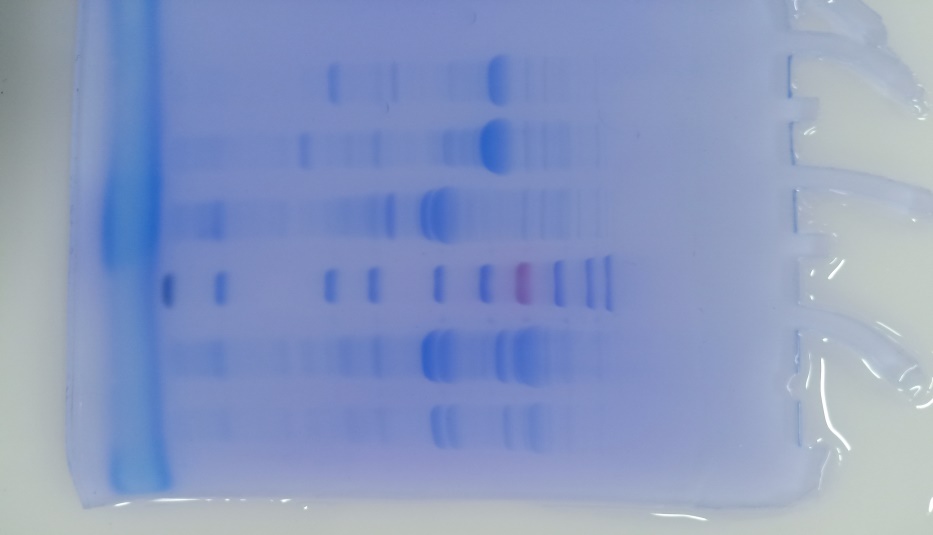


70

55

43

33

25

17

**FDC**

**PAD**

**SpEH**

**SMO(styA)**

**SMO(styB)**

kDa

1 2 3 M


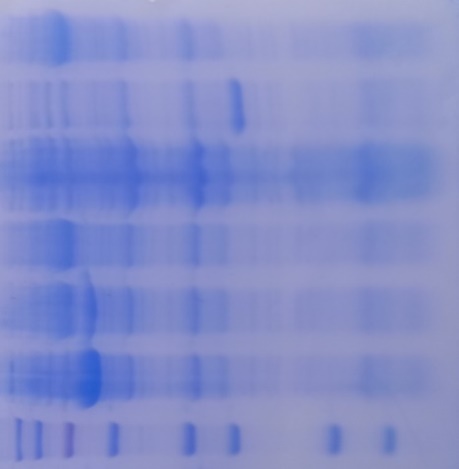


M 1

kDa

**FDC**

**PAD**

50

70

35

25

20

15

E

F

D


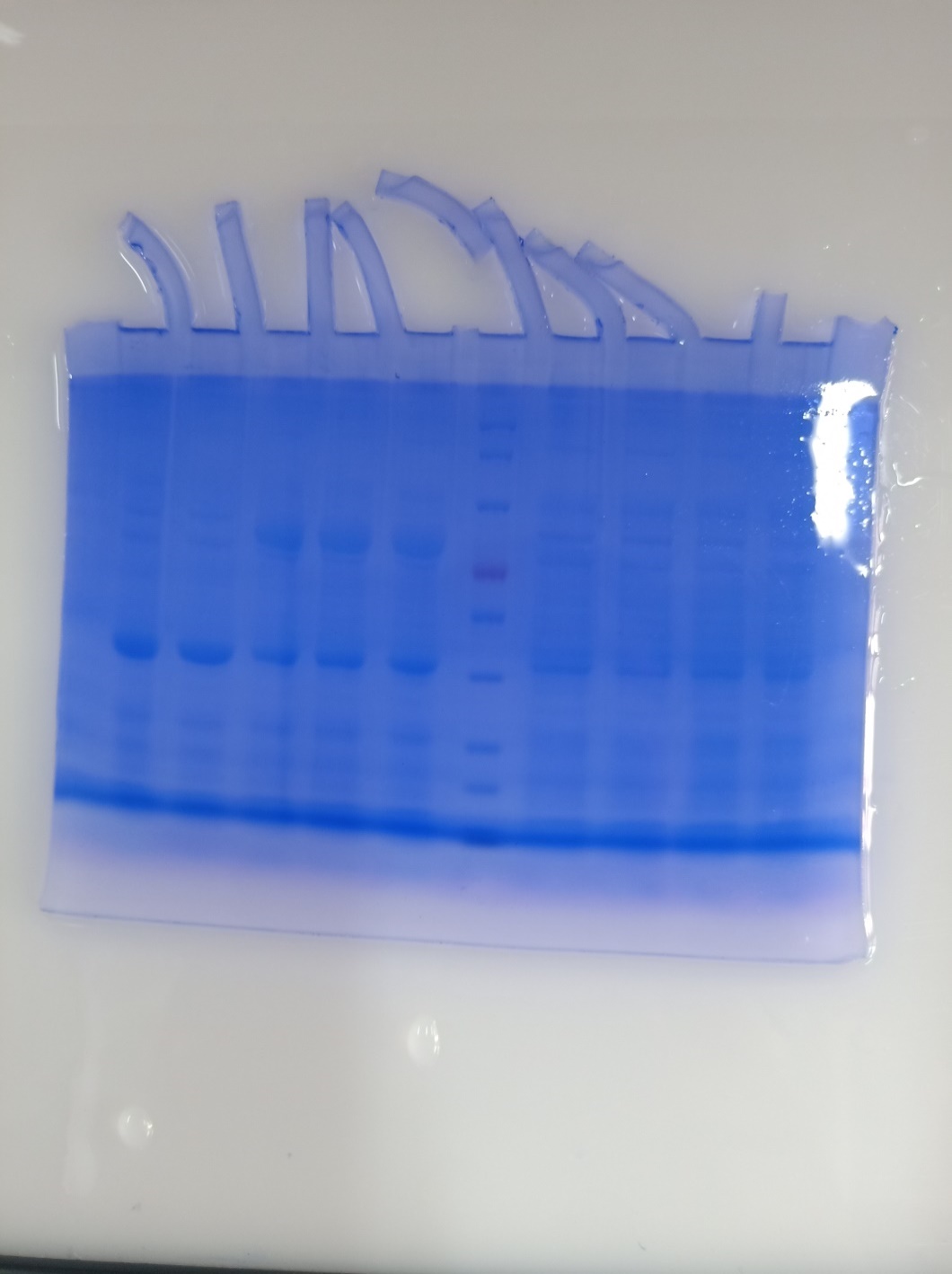


1 M

100

70

55

43

33

25

17

**PAL**

**SMO(styA)**

**SMO(styB)**

kDa

G

**Figure S3.** SDS-PAGE of the cell-free extracts of recombinant *E. coli* cells co-expression of multiple enzymes. A: laneM：marker , lane1,2：*E. coli* (DEA), lane3,4：*E. coli* (CGS); B: laneM：marker, lane1,2：*E. coli* (REG); C: laneM：marker, lane1,2：*E. coli* (DFP); D: laneM：marker , lane1：*E. coli* (CFP); E: laneM：marker , lane1,2：E. coli (RFP), lane3：E. coli(DES); F: laneM：marker , lane1,2：E. coli (RGA); G: laneM：marker , lane1：E. coli (CAS) .

2-HAP

A

IS

2-HAP

B

IS

2-HAP

C

IS

2-HAP

D

**Figure S4.** Achiral GC chromatograms of 2-HAP. A: 2-HAP standard. B: 2-HAP produced by conversion of L-phenylalanine (10 mM) with resting cells of *E. coli* (RFP-DEA-CGS) (15 g cdw/L) at 3 h. C: 2-HAP produced by conversion of L-phenylalanine (20 mM) with resting cells of *E. coli* (RFP-DEA-CGS) (25 g cdw/L) at 9 h. D: 2-HAP produced by conversion of L-phenylalanine (50 mM) with resting cells of *E. coli* (RFP-DEA-CGS) (25 g cdw/L) at 12 h. IS: Internal standard (*n*-dodecane), 4.05 min; 2-HAP: 2-hydroxyacetophenone, 4.91 min;


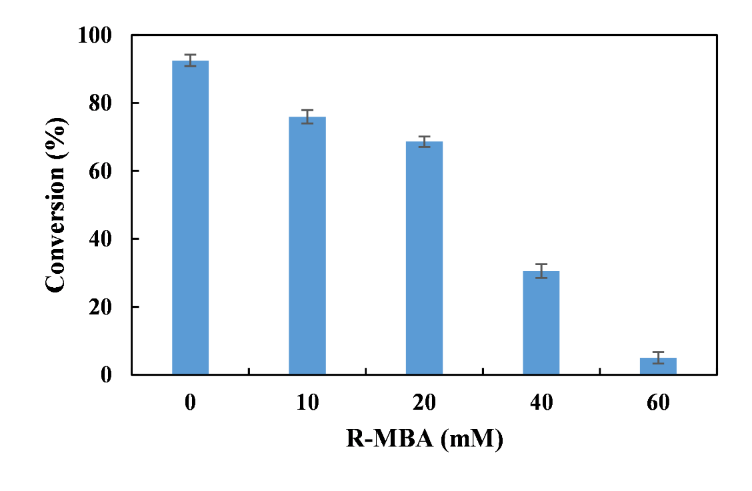


**Figure S5.** Effect of different concentration of R-MBA on *E. coli* (RFP-DEA-CGS) cells for conversion of L-PA to 2-HAP.

(*S*)-phenylglycinol

A

AP

R-MBA

IS

(*S*)-phenylglycinol

B

AP

R-MBA

IS

(*S*)-phenylglycinol

C

**Figure S6**. Achiral GC chromatograms of (*S*)-phenylglycinol. A: (*S*)-phenylglycinol standard. B: (*S*)-phenylglycinol produced by conversion of L-phenylalanine (10 mM) with resting cells of *E. coli* (RFP-DEA-CGS) (20 g cdw/L) and *E. coli* (MVTA) (15 g cdw/L) at 12 h. C: (*S*)-phenylglycinol produced by conversion of L-phenylalanine (20 mM) with resting cells of *E. coli* (RFP-DEA-CGS) (20 g cdw/L) and *E. coli* (MVTA) (15 g cdw/L) at 12 h. IS: Internal standard (*n*-dodecane), 4.05 min; R-MBA: (*R*)-(+)-1-phenylethylamine, 2.62 min; AP: acetophenone, 2.78 min; Product (*S*)-phenylglycinol, 6.05 min.

(*R*)-phenylglycinol

A

IS

(*R*)-phenylglycinol

B

IS

(*R*)-phenylglycinol

C

**Figure S7.** Achiral GC chromatograms of (*R*)-phenylglycinol. A: (*R*)-phenylglycinol standard. B: (*R*)-phenylglycinol produced by conversion of L-phenylalanine (10 mM) with resting cells of *E. coli* (RFP-DEA-CGS) (15 g cdw/L) and *E. coli E. coli* (EB-DGA) (20 g cdw/L) at 9 h. C: (*R*)-phenylglycinol produced by conversion of L-phenylalanine (20 mM) with resting cells of *E. coli* (RFP-DEA-CGS) (15 g cdw/L) and *E. coli* (EB-DGA) (20 g cdw/L) at 12 h. IS: Internal standard (*n*-dodecane), 4.05 min; (*R*)-phenylglycinol, 6.05 min.

.

(*S*)-phenylglycinol

(*R*)-phenylglycinol

A

(*R*)-phenylglycinol

B

(*R*)-phenylglycinol

(*S*)-phenylglycinol

D

C

(*S*)-phenylglycinol

E

**Figure S8**. Chiral GC chromatograms of phenylglycinol. A: (±)- phenylglycinol standard. B: (*R*)-phenylglycinol standard. C: (*S*)-phenylglycinol standard. D: (*R*)-phenylglycinol produced by conversion of L-phenylalanine (20 mM) with resting cells of *E. coli* (RFP-DEA-CGS) (15 g cdw/L) and *E. coli* (EB-DGA) (20 g cdw/L) at 12 h. E: (*S*)-phenylglycinol produced by conversion of L-phenylalanine (20 mM) with resting cells of *E. coli* (RFP-DEA-CGS) (20 g cdw/L) and *E. coli* (MVTA) (15 g cdw/L) at 9 h. (*R*)-phenylglycinol, 28.7 min; (*S*)-phenylglycinol, 29.3 min.

(*S*)-phenylglycinol

(*R*)-phenylglycinol

A

(*R*)-phenylglycinol

B

(*R*)-phenylglycinol

(*S*)-phenylglycinol

D

C

(*S*)-phenylglycinol

E

**Figure S9**. Chiral GC chromatograms of phenylglycinol. A: (±)- phenylglycinol standard. B: (*R*)-phenylglycinol standard. C: (*S*)-phenylglycinol standard. D: Preparation of (*R*)-phenylglycinol from L-phenylalanine. E: Preparation of (*S*)-phenylglycinol from L-phenylalanine. (*R*)-phenylglycinol, 28.7 min; (*S*)-phenylglycinol, 29.3 min.

**Figure S10.** ^1^H NMR spectra analysis of **7**.

**References**

[1] Xu Y, Jia X, Panke S, Li Z (2009) Asymmetric dihydroxylation of aryl olefins by sequential enantioselective epoxidation and regioselective hydrolysis with tandem biocatalysts. Chem Commun 1481–1483.

[2] Wu S, Li A, Chin YS, Li Z (2013) Enantioselective hydrolysis of racemic and meso-epoxides with recombinant *Escherichia coli* expressing epoxide hydrolase from *Sphingomonas* sp. HXN-200: preparation of epoxides and vicinal diols in high *ee* and high concentration. ACS Catal 3 (4): 752–759.

[3] Cui ZM, Zhang JD, Fan XJ, Zheng GW, Chang HH, Wei WL (2017) Highly efficient bioreduction of 2-hydroxyacetophenone to (*S*)- and (*R*)-1-phenyl-1,2-ethanediol by two substrate tolerance carbonyl reductases with cofactor regeneration. J Biotechnol 243: 1–9.

[4] Zhang JD, Zhao JW, Gao LL, Chang HH, Wei WL, Xu JH (2019a) Enantioselective synthesis of enantiopure β-amino alcohols via kinetic resolution and asymmetric reductive amination by a robust transaminase from *Mycobacterium vanbaalenii*. J Biotechnol 290: 24-32.

[5] Zhang JD, Yang XX, Dong R, Gao LL, Li J, Li X, Huang SP, Zhang CF, Chang HH (2020) Cascade biocatalysis for regio- and stereoselective aminohydroxylation of styrenyl olefins to enantiopure arylglycinols. ACS Sustainable Chem Eng 8 (49): 18277-18285.
